# Multifaceted Assessment of Amazonian Tree Diversity Reveals Pervasive Impacts of Human Modification

**DOI:** 10.1111/gcb.70595

**Published:** 2025-11-10

**Authors:** Erika Berenguer, Cássio Alencar Nunes, Jesús Aguirre‐Gutiérrez, Joice Ferreira, Yadvinder Malhi, Luiz E. O. C. Aragão, Adriane Esquivel‐Muelbert, Axa E. S. Figueiredo, Joseph E. Hawes, Carlos A. Joly, Carlos A. Quesada, Marina M. M. de Seixas, Ima Vieira, Jos Barlow

**Affiliations:** ^1^ Lancaster Environment Centre Lancaster University Lancaster UK; ^2^ Environmental Change Institute University of Oxford Oxford UK; ^3^ Ecologia Aplicada, Departamento de Ecologia e Conservação Universidade Federal de Lavras Lavras Brazil; ^4^ Leverhulme Centre for Nature Recovery University of Oxford Oxford UK; ^5^ Embrapa Amazônia Oriental Belém Brazil; ^6^ Earth Observation and Geoinformatics Division National Institute for Space Research (INPE) São José dos Campos Brazil; ^7^ College of Life and Environmental Sciences University of Exeter Exeter UK; ^8^ Department of Plant Sciences University of Cambridge Cambridge UK; ^9^ Coordination of Environmental Dynamics National Institute for Amazonian Research Manaus Brazil; ^10^ Institute of Science and Environment University of Cumbria Ambleside UK; ^11^ Departamento de Biologia Vegetal, Instituto de Biologia Universidade Estadual de Campinas Campinas Brazil; ^12^ Museu Paraense Emílio Goeldi Belém Pará Brazil

**Keywords:** Amazon, biodiversity, degradation, dominance, fire, hill numbers, human‐modified‐tropical forests, logging, secondary forests, trees

## Abstract

Tropical forests harbour the majority of tree species on the planet but are increasingly subjected to deforestation and human‐driven disturbances. Understanding how human modifications impact various facets of diversity—i.e., taxonomic, functional, and phylogenetic—is crucial, as their responses can differ significantly. Additionally, the influence of species dominance and individual size class on the recovery trajectories of future forests is often overlooked. Here, we address these knowledge gaps by comparing the taxonomic, functional, and phylogenetic diversities of large (≥ 10 cm DBH) and small (≤ 2 cm DBH < 10 cm DBH) trees in undisturbed and human‐modified Amazonian forests, considering different weights of species dominance using Hill Numbers. We sampled 25,313 large and 30,070 small trees across 215 forest plots distributed in two different regions of Eastern Amazonia and representing a range of human modification (i.e., undisturbed, logged, logged‐and‐burned, and secondary forests). Our findings indicate that human modifications significantly reduce the taxonomic, functional, and phylogenetic diversities of both large and small trees, regardless of dominance weightings. Secondary forests exhibited the lowest alpha‐diversity and were the most dissimilar to undisturbed forests, while logged‐and‐burned forests were as distinct from undisturbed forests as they were from secondary forests across all diversity facets. Taxonomic and functional diversity showed similar sensitivity to human modification, while phylogenetic diversity was the least sensitive in alpha‐diversity but equally sensitive in community composition analyses. Overall, we showed that human modification explained 55% of the effect size variation found in alpha‐diversity and 42% of that found in community composition, with diversity facet, tree size and dominance weighting explaining either ≤ 5%. Given the deleterious impacts of human modification on the diversity of tropical forests, it is imperative to protect remaining undisturbed areas from selective logging and wildfires. Nevertheless, even disturbed primary forests still harbour more taxonomic, functional and phylogenetic diversity than secondary forests.

## Graphical abstract

Amazonian forests impacted by humans have a reduced and different group of tree species than those forests that have never been impacted. The trees present in forests impacted by humans also have different evolutionary histories and functional characteristics. It is essential to protect the remaining forests from further disturbances to avoid biodiversity loss in Amazonian forests.

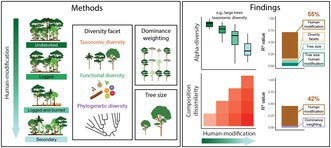

## RESUMO

As florestas tropicais abrigam a maioria das espécies de árvores do planeta, mas estão cada vez mais sujeitas ao desmatamento e a distúrbios causados por atividades humanas. Compreender como as modificações antrópicas afetam diferentes facetas da biodiversidade—isto é, taxonômica, funcional e filogenética—é fundamental, uma vez que suas respostas podem diferir significativamente. Além disso, a influência da dominância das espécies e da classe de tamanho dos indivíduos nas trajetórias de recuperação das florestas é frequentemente negligenciada. Neste estudo, abordamos essas lacunas de conhecimento comparando as diversidades taxonômica, funcional e filogenética de árvores grandes (DAP ≥ 10 cm) e pequenas (2 cm ≤ DAP < 10 cm) em florestas amazônicas intactas e aquelas modificadas por atividades humanas, considerando diferentes ponderações de dominância de espécies por meio dos Números de Hill. Para isso, nós amostramos 25.313 árvores grandes e 30.070 árvores pequenas em 215 parcelas florestais distribuídas em duas regiões distintas da Amazônia Oriental, representando diferentes níveis de modificação antrópica (incluindo florestas intactas, com exploração de madeira, com exploração de madeira e queimadas, e florestas secundárias). Nossos resultados indicam que as modificações humanas reduzem significativamente as diversidades taxonômica, funcional e filogenética, tanto de árvores grandes quanto pequenas, independentemente das ponderações de dominância. As florestas secundárias apresentaram a menor diversidade alfa e foram as mais distintas das florestas intactas, enquanto as florestas exploradas e queimadas mostraram‐se tão diferentes das florestas intactas quanto das florestas secundárias em todas as facetas de diversidade. A diversidades taxonômica e funcional exibiram sensibilidade semelhante às modificações humanas, enquanto a diversidade filogenética foi a menos sensível em termos de diversidade alfa, mas igualmente sensível nas análises de composição das comunidades. De modo geral, demonstramos que as modificações humanas explicaram 55% da variação no tamanho do efeito observado na diversidade alfa e 42% daquela observada na composição das comunidades, enquanto a faceta da diversidade, o tamanho das árvores e a ponderação por dominância explicaram ≤ 5%. Diante dos impactos deletérios das modificações humanas sobre a diversidade das florestas tropicais, é imperativo proteger as áreas intactas remanescentes contra a exploração seletiva e os incêndios florestais. Ainda assim, as florestas primárias degradadas mantêm maior diversidade taxonômica, funcional e filogenética do que as florestas secundárias.

## 1 Introduction

Tropical rainforests harbour the majority of the world's biodiversity (Barlow et al. 2018; Fine and Ree 2006), making their conservation pivotal for slowing down the current biodiversity crisis (Díaz et al. 2019). However, in the 21st century, forest loss has been higher in the tropics than anywhere else in the world (Harris et al. 2021). Forest loss is not the only threat to tropical forests—human disturbances, such as selective logging and fires are pervasive (Pearson et al. 2017). Across Amazonia, for example, the area affected by these disturbances every year is greater than that deforested (Lapola et al. 2023). Currently, human‐modified Amazonian forests, which include both disturbed primary forests and secondary forests (i.e., those growing in areas that have been completely deforested before), occupy at least 1.27 million km^2^, representing 23% of the remaining forests in the region (Bullock et al. 2020; Smith et al. 2021). Despite undisturbed primary forests being irreplaceable for sustaining biodiversity (Gibson et al. 2011), holding richer communities than those found in human‐modified forests, the role of the latter is becoming increasingly important as, in many landscapes, they are the last stronghold of a vanishing ecosystem (Gaveau et al. 2016; Guedes Pinto et al. 2023; Malhi et al. 2014; Ribeiro et al. 2009).

Changes in taxonomic diversity in human‐modified tropical forests are well documented, with studies showing local and regional extinctions of several taxa, from birds and mammals to insects and plants (Barlow et al. 2007; López‐Bedoya et al. 2022; Martin et al. 2013). Local extinctions of species that are dependent on undisturbed forests are usually followed by a sharp increase in pioneer or generalist species, resulting in significantly different compositions between pre‐ and post‐disturbance communities (Filgueiras et al. 2021; Lohbeck et al. 2016; Pinho et al. 2025). For example, burned Amazonian forests, when compared to undisturbed ones, experienced an increase in abundance of 1680% of a single pioneer species (da Silva et al. 2020). Human modifications also bring about changes in the distribution of key functional traits (Both et al. 2019; Hogan et al. 2018), affecting forests' functional diversity (Ernst et al. 2006; Hawes et al. 2020; Mestre et al. 2020) and impacting a number of ecosystem functions, such as seed dispersal and the fixation of soil nitrogen (Reiss et al. 2009; Wong et al. 2020). Human modifications can also impact the phylogenetic composition of affected forests if the resulting species turnover generates phylogenetic clustering—i.e., communities with less divergent evolutionary histories (Ding et al. 2012).

Although these three facets of diversity (i.e., taxonomic, functional, and phylogenetic) can be affected by human modification, understanding their different responses and sensitivities is important as responses are not always congruent (Devictor et al. 2010; Mazel et al. 2018) and they provide different and complementary information on forests' resilience (Aguirre‐Gutiérrez et al. 2020; Winter et al. 2013). For instance, while taxonomic diversity (TD) will measure and indicate the identity and richness of species, functional diversity (FD) will reflect the diversity of a multitude of traits (e.g., morphological, chemical., reproductive) that have a closer linkage with ecosystem functioning, with higher FD indicating higher functional stability (Cadotte et al. 2011). On the other hand, phylogenetic diversity (PD) captures the relationship of biogeographic histories and lineage diversity with community structure and will indicate the ability of a community to adapt to environmental changes (Cadotte et al. 2012; Forest et al. 2007; Srivastava et al. 2012). By analysing these three facets together, we can create a more holistic understanding of communities in human‐modified ecosystems, possibly uncovering patterns that would not be detected if only taxonomic diversity was evaluated. This was the case, for example, for arboreal ants in New Guinea, which presented higher taxonomic diversity in primary forests, but higher functional diversity in secondary ones, likely reflecting high competition levels among species colonizing this new habitat (Hoenle et al. 2024).

Despite the ubiquity of human‐modified rainforests across the tropics, there is a paucity of research investigating how these modifications affect all three diversity facets e.g., (López‐Baucells et al. 2022; Rurangwa et al. 2021). Furthermore, the majority of studies looking at the impacts of human modification on diversity focus on occurrence (presence/absence) and do not address the changes in species dominance, which are so prominent in human‐modified forests (Filgueiras et al. 2021). Finally, for indeterminate growers (i.e., organisms that grow throughout their lives), such as plants, the different size classes of a community are seldom analysed separately, with most tropical forest studies focusing only on large trees—i.e., individuals ≥ 10 cm diameter at breast height (DBH) (e.g., Aguirre‐Gutiérrez et al. 2020; Esquivel‐Muelbert et al. 2019; Marca‐Zevallos et al. 2022). However, small trees—i.e., individuals < 10 cm DBH—are known to respond faster to human modification than large ones (Krishnadas et al. 2019; Rocha‐Santos et al. 2023; Slik et al. 2002), due to lagged recruitment i.e., a tree may take centuries until it recruits into the 10 cm size class (Vieira et al. 2005). This could possibly result in significant differences in the taxonomic, functional and phylogenetic composition of different size classes, which could potentially be reflected in the future of the forest stand (Berenguer et al. 2018).

Here, we address these knowledge gaps by comparing the taxonomic, functional and phylogenetic diversities of large and small trees in undisturbed and human‐modified Amazonian forests, while considering different metrics of species dominance (i.e., Hill Numbers, with different weights attributed to rare, common, and dominant species—see Methods for more details). We use data from 215 plots distributed across undisturbed, logged, logged‐and‐burned, and secondary forests in two Amazonian regions where human modification is prevalent across the landscape. Our sampling encompassed 25,313 large and 30,070 small trees, which we combined with data on 20 functional traits, 14 of which were sampled in situ. Specifically, we ask: (Q1) How does human modification affect the diversity of large and small trees? (Q2) Which facets of diversity and which tree size class are most sensitive to human modification? We address these questions by focusing on the alpha‐diversity and community composition of the sampled plots.

## 2 Methods

### 2.1 Study Areas

**FIGURE 1:**
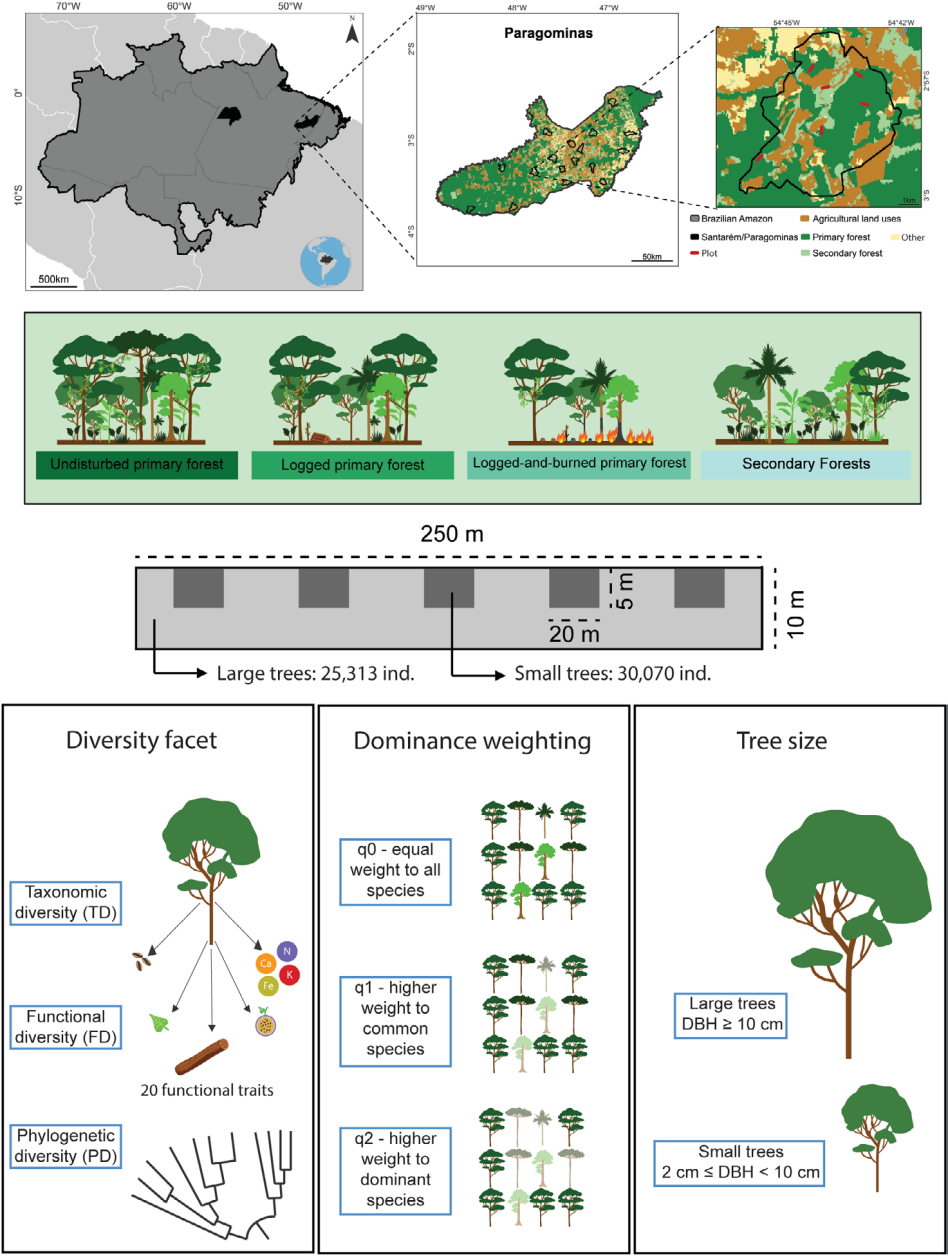
Sampling design. Map of the study area in the municipalities of Santarém and Paragominas (in black) in the state of Pará (grey contour) in the Brazilian Amazon (black contour). The sampling of large trees (i.e., ≥ 10 cm DBH) was conducted in 215 plots of 10 × 250 m. Small trees (i.e., 2–9.9 cm DBH) were sampled in five 20 × 5 m subplots within each plot. We obtained the alpha diversity and the dissimilarity in species composition in four forest classes, being 1—Undisturbed, 2—Logged and 3—Logged‐and‐burned primary forests, and 4—Secondary forests. For each plot, we used the Hill Numbers approach to calculate three facets of diversity (Taxonomic—TD, Functional—FD, and Phylogenetic—PD diversities), and used three dominance weightings to access the importance of relative abundance of the species in diversity calculation (parameter *q*). With *q* = 0, there is no weight for species abundance and diversity is the same as species richness, with *q* = 1, common species have more weight in the diversity calculation, similar to Shannon Index, and with *q* = 2, the weight of dominant species is higher, similar to Gini‐Simpson index. Map lines delineate study areas and do not necessarily depict accepted national boundaries.

We sampled two regions of Eastern Amazonia—Paragominas and Santarém (the latter including also the municipalities of Belterra and Mojuí dos Campos; Figure 1). In each region, we selected 18 third‐order catchments with an average of 5000 ha each (see Gardner et al. 2013 for more details). In each catchment, we established plots (250 × 10 m) in evergreen non‐flooded forests, located at least 100 m away from forest edges. Plots were separated by a minimum distance of 1500 m to avoid spatial autocorrelation. The number of plots per catchment varied according to the proportion of forest cover present—i.e., catchments with more forest had more plots than those with less forest, maintaining a standard density of one plot per 400 ha. In total, we sampled 215 plots distributed across four forest classes: undisturbed, logged, logged‐and‐burned, and secondary forests (Figure 1, Table S1). Each forest class was determined using a combination of on‐the‐ground assessments of past human modification (e.g., the presence of charcoal or logged stumps) with the visual analysis of a chronosequence of Landsat images covering 22 years in the case of Paragominas and 20 years for Santarém (more details in Berenguer et al. 2014).

### 2.2 Large and Small Tree Sampling

Across all 215 plots, we sampled all trees ≥ 10 cm diameter at breast height (DBH). In each plot, we set five subplots (20 × 5 m) in which we sampled all trees ≥ 2 cm DBH < 10 cm DBH (Figure 1). From the 55,383 sampled individuals, 475 could not be identified to species level and were excluded from all analyses.

### 2.3 Functional Trait Sampling

We compiled data from 20 functional traits (Table S2)—14 of which were sampled in situ in Santarém and six from data published in the literature. In a subsample of our 215 plots (*n* = 21), we sampled bark thickness in all individuals ≥ 10 cm DBH, totaling 2470 trees from 426 species (see Berenguer et al. 2021 for more information). Leaf area, leaf dry matter content, leaf thickness, specific leaf area and nine chemical traits (i.e., C, Ca, Fe, K, Mg, Mn, N, P, Zn) were sampled in 20 plots. For these traits, sampling focused only on the species that contributed to 80% of the basal area of each plot (more details in Berenguer et al. 2021). From each sampled individual, a branch that was fully exposed to the sun was collected. In total, these traits were sampled in 1325 individuals from 260 species. Dispersal traits (i.e., seed width, fruit type, and dispersal mode) were compiled from herbarium samples combined with several sources in the literature (see Hawes et al. 2020 for a complete list of data sources). Potential tree size and water deficit affiliation were derived from data from > 500 plots distributed across Amazonia (the sampling approach is described in Esquivel‐Muelbert et al. 2019). Finally, wood density was compiled from the Wood Density Database (Zanne et al. 2009), filtering by tropical South America. When a given trait was available for multiple individuals of the same species, we averaged it at the species level. For species lacking any trait information, we attributed genus‐level data; when that was not possible, we assigned family‐level data (Tables S3 and S4). We excluded from these analyses five individuals from three species, which only had data for a single trait. Finally, we performed pairwise correlation analysis between all continuous traits using the function “cor” in R software. None of the trait pairs had a strong correlation (i.e., all *r* < 0.7; Figure S1).

### 2.4 Phylogenetic Tree Construction

We constructed a single phylogenetic tree for all our dataset (i.e., including all species from both sampled regions, Figure S2). For this, we used the R20100701 ultrametric tree from Phylomatic (Webb and Donoghue 2005), where the branch lengths were adjusted using the default ages file (Wikström et al. 2001). Based on the resulting tree we calculated the mean pairwise phylogenetic distance (Webb et al. 2008), using a null model based on frequency by randomizing the within‐species community abundance. We excluded 500 individuals of 27 species that were not present in the phylogenetic tree from the analyses of this study.

### 2.5 Data Analysis

#### 2.5.1 Alpha‐Diversity and Differences in Community Composition Calculation

We used the Hill numbers approach to calculate alpha‐diversity and differences in community composition (Chao et al. 2014). Hill numbers provide a framework where biological diversity is measured as the effective number of taxonomic, functional and phylogenetic entities, which, in turn, can be weighted by some measure of relative importance, such as abundance (Chao et al. 2014). The parameter q determines the weight we want to give to relative abundances in an assemblage. With *q* = 0, the relative abundance of each species is not considered, and for instance, the effective number of taxonomic entities equals the number of species (i.e., the same as species richness). With *q* = 1 the species are weighted by their frequencies, and Hill numbers are the effective number of common species in the assemblage, a conversion from and thus theoretically similar to the Shannon Index. Finally, with *q* = 2, very abundant species receive a higher weight and Hill numbers are the effective number of dominant species in a given assemblage, a conversion from and thus theoretically similar to the Gini‐Simpson index.

To calculate alpha‐diversity for the three diversity facets (i.e., taxonomic, functional, and phylogenetic) we used the “iNEXT.3D” package in R (Chao et al. 2021), with three different dominance weightings (i.e., *q*0, *q*1 and *q*2) to obtain alpha taxonomic, functional and phylogenetic diversities for each of the sampled plots, for both large and small trees. For community composition, we calculated a dissimilarity matrix for each diversity facet, for each dominance weighting, for large and small trees and for each sampling region separately. For this, we used the “iNEXT.beta3D” package in R (Chao et al. 2023). In both cases (i.e., alpha‐diversity and community composition), we used matrices of species × plots with species abundances in cells to calculate taxonomic alpha‐diversity and differences in community composition. For functional diversity, we first used values for the 20 functional traits of all species to construct species functional distance matrices based on the Gower distance, using the package “FD” (Laliberté et al. 2023). We did this separately for species of large and small trees and for Paragominas and Santarém. We used these distance matrices along with species × plots matrices to calculate functional diversity (Figure S2). Similarly, for phylogenetic diversity, we cut the global phylogenetic tree of all species into four phylogenetic trees for large and small trees and for each sampling region, and used it to calculate phylogenetic alpha‐diversity and differences in community composition for each plot (Figure S2).

#### 2.5.2 Effect of Human Modification on Alpha‐Diversity and Community Composition

To evaluate the impacts of human modification on alpha diversity, we first scaled and centered the values of alpha diversity. Then, we used the scaled alpha diversity to run linear mixed effect models (LMMs) separately for each region, each tree size, each diversity facet and each dominance weighting, ending up with 36 models (2 regions × 2 tree sizes × 3 diversity facets × 3 dominance weightings). In all models, we considered forest class as an explanatory variable and included catchment as a random factor (Figure S2). We ran a contrast analysis for each model to understand which forest classes were different from each other. To do that, we used the “multcomp” package and corrected *p* values for multiple comparisons using the “single‐step” method (Hothorn et al. 2008).

For community composition, we used the dissimilarity matrices of taxonomic, functional and phylogenetic diversity of large and small trees calculated using the three dominance weightings and for both regions to run PERMANOVAs. As for alpha‐diversity, we ran 36 PERMANOVAs, including forest class and catchment as explanatory variables using the “vegan” package (Oksanen et al. 2022). After that, to test which forest class had different community composition from each other, we ran pairwise PERMANOVAs using the “pairwiseAdonis” package (Martinez Arbizu 2020). The *p* values were adjusted using the “bonferroni” method. To visualize the dissimilarities in community composition, we ran a Non‐Metric Multidimensional Scaling (NMDS) analysis for each dissimilarity matrix (36 in total) using the vegan package in R (Oksanen et al. 2022).

#### 2.5.3 Sensitivity of Different Facets of Diversity, Dominance Weightings and Tree Sizes to Human Disturbances

To evaluate the sensitivity of different metrics, we first calculated the effect sizes of each forest class comparison for each metric. As we scaled and centered the alpha‐diversity to run LMMs, we used the differences in estimates for each pair of forest class comparisons as our effect size metric. We used the “multcomp” package (Hothorn et al. 2008) to run the pairwise comparisons (as done in the contrast analysis described in section 2.5.2) resulting in six effect size values for each LMM (6 × 36 = 216 in total). For community composition, as *R* ^2^ values are biased by the number of degrees of freedom, we calculated Omega^2^ for each comparison of forest class and for each metric from the pairwise PERMANOVAs. Omega^2^ values are unbiased estimators of effect sizes and can also be interpreted as variance explained.

To understand the sensitivity of different diversity facets, dominance weightings and tree sizes to human modifications, we ran two LMMs (one for alpha‐diversity and one for community composition) using effect sizes as response variables and a four‐way interaction as an explanatory variable (Figure S2). The four‐way interaction combined the pair of forest class comparison, diversity facet, dominance weighting and tree size. In addition, we used region as a random factor. We followed a method of backward stepwise elimination of non‐significant terms to obtain a minimum model that best explained the variation in effect sizes. After we obtained the minimum model, we ran contrast analysis to verify which levels of each factor were different from each other using the “emmeans” package (Lenth 2023). Finally, we calculated the variance explained by each term (variance partition) of the minimum models using the “partR2” package (Stoffel et al. 2021).

## 3 Results

### 3.1 Effects of Human Disturbances on Alpha‐Diversity and Community Composition

**FIGURE 2:**
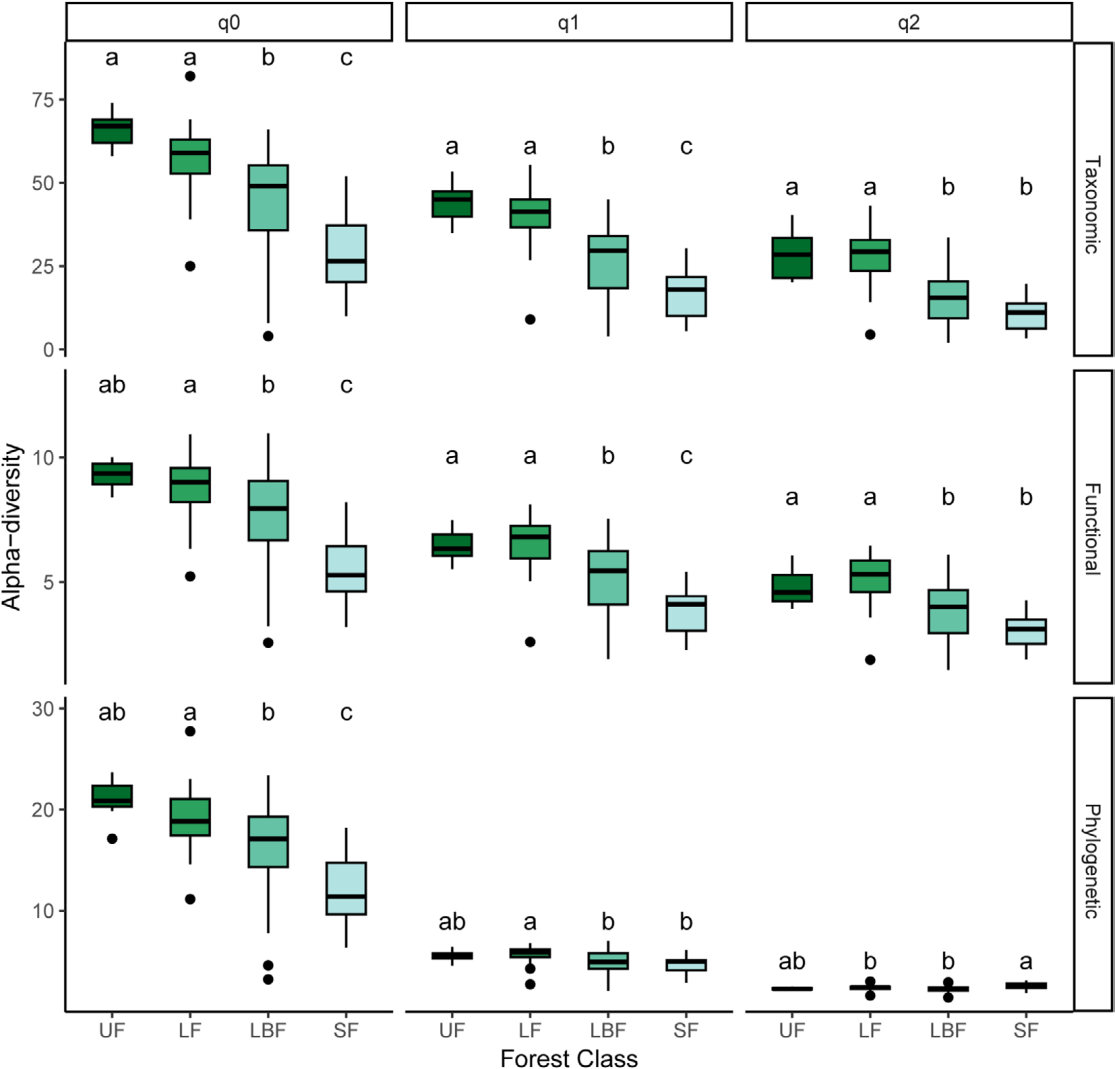
Alpha‐diversity of large trees sampled in four forest classes in Paragominas, in the Brazilian Amazon. Alpha‐diversity was calculated for each dominance weighting (i.e., *q*0, *q*1, and *q*2) and diversity facet. The Y axes for taxonomic, functional and phylogenetic diversity have different scales. Different letters denote significant differences (*p* < 0.05) in mean values within each dominance weighting and facet analysed. LBF, logged‐and‐burned forests; LF, logged forests; SF, secondary forests; UF, undisturbed forests. Results for small trees in Paragominas, and large and small trees in Santarém can be found in the Figures S3–S5.

**FIGURE 3:**
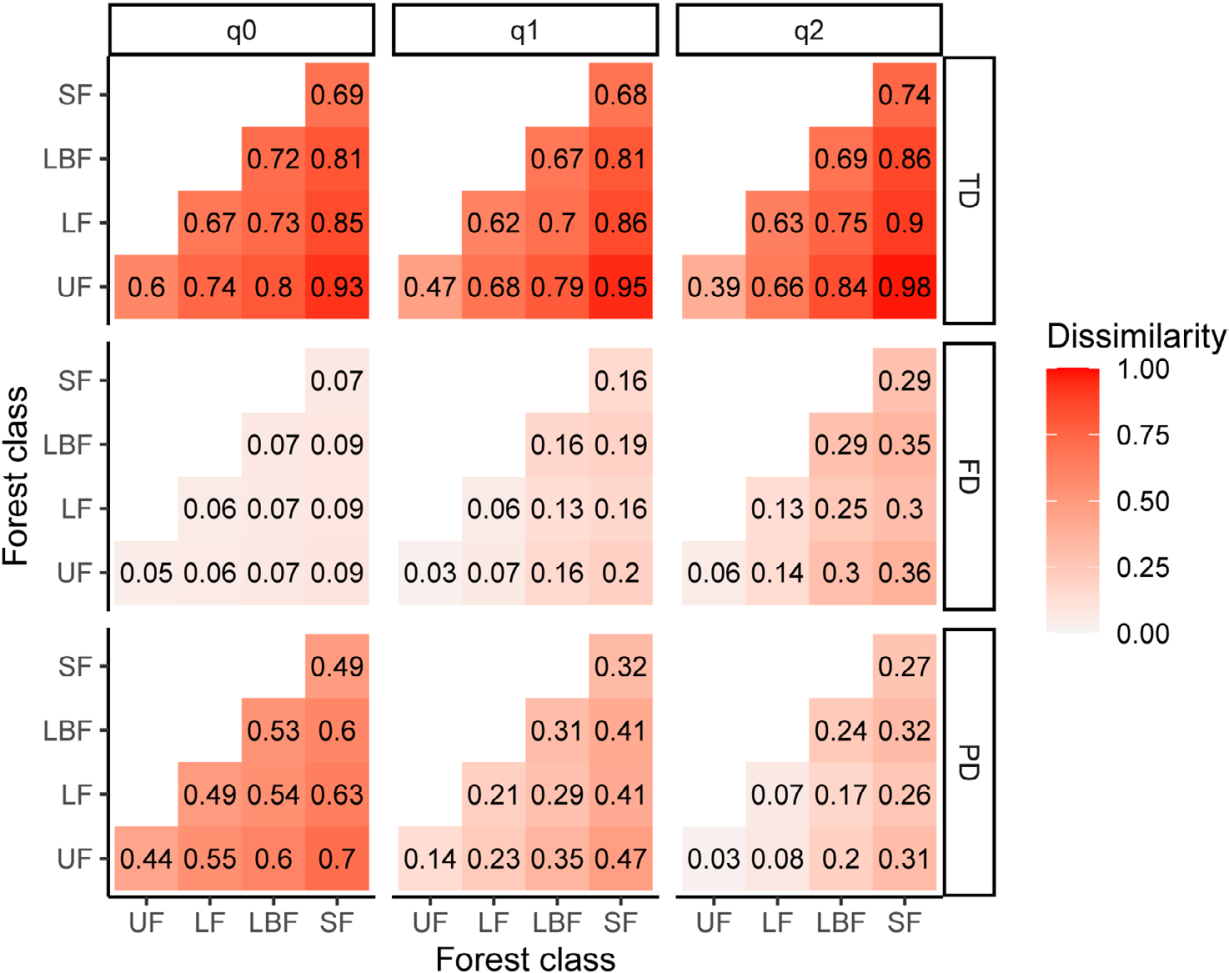
Mean pairwise dissimilarity in community composition of large trees sampled in four forest classes in Paragominas, in the Brazilian Amazon. Dissimilarity was calculated using different dominance weightings (i.e., *q*0, *q*1 and *q*2) for taxonomic, functional, and phylogenetic diversity. LBF, logged‐and‐burned forests; LF, logged forests; SF, secondary forests; UF, undisturbed forests. Results for small trees in Paragominas, and large and small trees in Santarém can be found in the Figures S6–S8.

Human modifications affected most of the alpha‐diversity and all the composition models—i.e., at least two forest classes were significantly different from each other for 33 of the 36 models assessing changes in alpha‐diversity and for all the 36 models assessing changes in composition (Table 1). In terms of pairwise comparisons, secondary forests generally showed lower taxonomic, functional and phylogenetic alpha‐diversity when compared to all classes of primary forests, representing the forest class with the highest number of significant differences in relation to the others (Figure 2 and Figures S3–S5). Mirroring the pattern found for alpha‐diversity, community composition of secondary forests presented the highest number of significant differences when compared to all individual classes of primary forests (Table 1), showing the highest values of dissimilarity (Figure 3 and Figures S6–S12).

**TABLE 1:** Number of times a forest class was significantly different from another following alpha‐diversity (i.e., 36 LMMs) and composition (i.e., 36 PERMANOVAs) analyses.

Forest class	Undisturbed	Logged	Logged‐and‐burned
Alpha‐diversity			
Undisturbed	—		
Logged	1	—	
Logged‐and‐burned	5	21	—
Secondary	22	33	28
Composition			
Undisturbed	—		
Logged	18	—	
Logged‐and‐burned	26	31	—
Secondary	34	36	30

### 3.2 Sensitivity of Different Facets of Diversity, Dominance Weightings and Tree Sizes to Human Modifications

**FIGURE 4:**
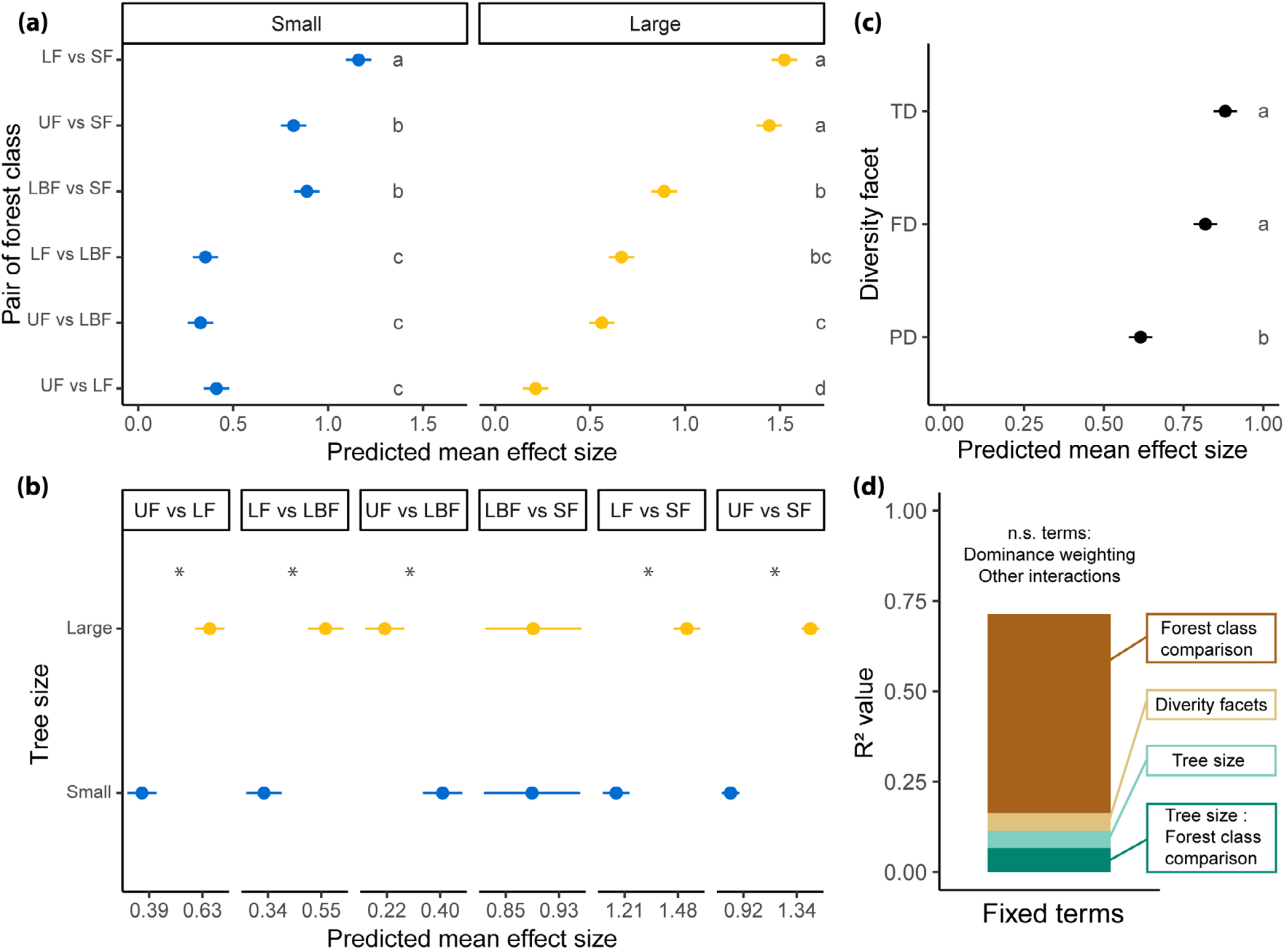
Sensitivity analysis of alpha‐diversity responses to human modifications. The predicted mean effect sizes and standard errors for differences in alpha‐diversity values are shown only for the significant terms of the sensitivity analysis, which were (a) forest class comparisons, (b) tree size and (c) diversity facet. Panels (a) and (b) display identical effect size values but are faceted by either forest class comparison or tree size (small = blue, large = yellow) to better illustrate the interaction between them. Different letters in (a) show forest class comparisons that have different effect sizes within each tree size class. Asterisks in (b) show forest class comparisons in which large and small trees differed in mean effect size. Different letters in (c) show the different effect sizes between diversity facets. Panel (d) shows the variance partition of the effect sizes according to the sensitivity analysis. For estimated marginal means of each level of these fixed terms see Tables S5–S8. Facets of diversity: TD, taxonomic diversity; FD, functional diversity; PD, phylogenetic diversity. Forest classes: UF, undisturbed forests; LF, logged forests; LBF, logged‐and‐burned forests; SF, secondary forests.

**FIGURE 5:**
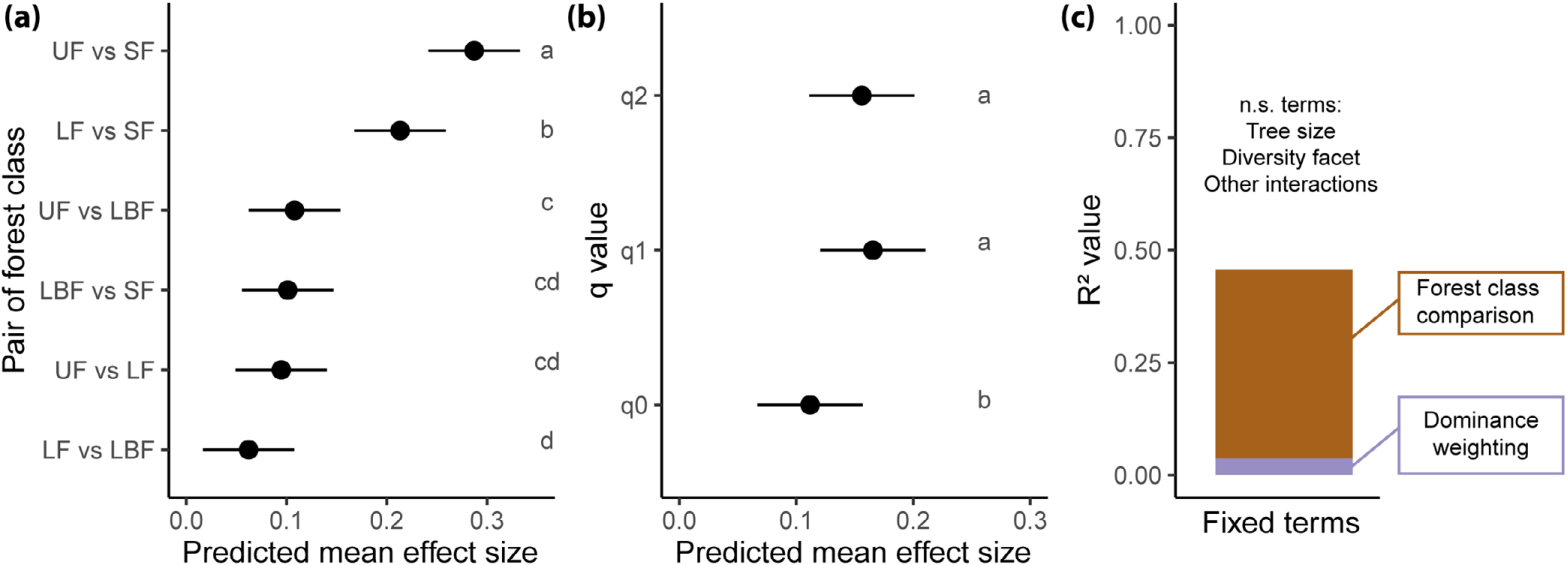
Sensitivity analysis of Amazonian tree community composition responses to human modifications. The predicted mean effect sizes and standard errors (Omega^2^ from PERMANOVAs) for differences in community composition are shown only for the significant terms of the sensitivity analysis, which were (a) forest class comparison and (b) dominance weightings (i.e., *q*0, *q*1 and *q*2). Letters indicate significant differences between forest class comparisons. Panel (c) shows the variance partition of the effect sizes according to the sensitivity analysis. Pairwise comparisons are ordered from highest to lowest mean effect sizes. For estimated marginal means of each level of these fixed terms see Tables S9–S11. UF, undisturbed forests; LF, logged forests; LBF, logged‐and‐burned forests; SF, secondary forests.

For alpha‐diversity, the taxonomic and functional facets were significantly more sensitive to human modifications than the phylogenetic one (Figure 4c, Table S5), while for community composition all three facets of diversity were equally sensitive. When looking at different dominance weightings (i.e., *q* values), we also found contrasting results between alpha‐diversity and community composition: while for the former there was no difference in the sensitivity of the different dominance weightings considered, for the latter *q*1 and *q*2 were significantly more sensitive than *q*0 (Figure 5b, Table S9). Overall, large trees were significantly more sensitive than small ones to human modifications (i.e., higher effect sizes) in alpha‐diversity analyses, although in the comparison between undisturbed and logged‐and‐burned forests, small trees were more sensitive, and between logged‐and‐burned and secondary forests there was no difference between the sensitivity of tree sizes (Figure 4a,b and Tables S6 and S7). For community composition, large and small trees were similarly sensitive. Across all models of alpha‐diversity, the highest effect sizes were observed in comparisons between the three classes of primary forests with secondary forests (Figure 4a and Table S8). For community composition, the pairwise comparisons between undisturbed and logged forests with secondary forests also showed the highest effect sizes; however, the comparison between logged‐and‐burned forests with secondary ones showed low effect sizes (Figure 5a and Table S10). Finally, we found that for alpha‐diversity 55% of the effect size variation was explained by the forest class comparison, while diversity facets and tree size explained only ~5% each, and dominance weighting was unimportant (Figure 4d). As with the alpha‐diversity analysis, forest class comparison explained most of the variation found in the models of community composition (42%), while dominance weightings explained only 3%, and diversity facets and tree size were unimportant (Figure 5c).

## 4 Discussion

Using a dataset of 55,383 individuals sampled across two different regions in Amazonia, combined with 20 functional traits and employing the most up‐to‐date phylogenetic tree available, we found that human modifications impacted the taxonomic, functional and phylogenetic diversity of both large and small trees, regardless of how dominance is accounted for in affected communities. In other words—logging, wildfires and clear felling have a much greater influence on plant diversity than the choice of diversity facet, tree size, or dominance weighting used in our analyses. This finding shows the profound effects of human modifications in Amazonian forests, and more specifically on the organisms that effectively constitute the structure of a forest and represent the primary energy source for a multitude of other taxa. The changes we demonstrate here go a long way to explaining why human‐modified forests also hold fauna communities that are taxonomically, functionally and phylogenetically different, and generally more impoverished, than those found in undisturbed forests (Colombo et al. 2023; Mestre et al. 2020; Solar et al. 2015). In summary, human‐modified forests are fundamentally different from their undisturbed counterparts. We discuss these results by first exploring what they reveal about the impact of different human modifications on diversity, before exploring the implications for how we measure and assess diversity.

### 4.1 Human Modification Negatively Affects Tree Diversity in Tropical Forests

Understanding the greatest drivers of diversity change in tropical forests is key to helping develop evidence‐based policies to prevent further biodiversity loss. We found that secondary forests had the lowest levels of alpha‐diversity and were the most dissimilar to undisturbed ones, regardless of the region, the diversity facet, the tree size or the dominance weighting analyzed. This result is hardly surprising as clear felling is the most severe form of human modification to a previously forested area. Secondary forests occupy as much as 235,000 km^2^ across Amazonia (Smith et al. 2021) and are the target of a growing wave of interest – as secondary forests grow, they sequester CO_2_ from the atmosphere, therefore making an essential contribution to mitigating climate change (Chazdon et al. 2016; Heinrich et al. 2021). However, secondary forests hold smaller carbon stocks than primary ones (Berenguer et al. 2014; Smith et al. 2020), and they also harbor a distinctive flora, which is much poorer taxonomically, functionally and phylogenetically. Restoration projects must be clear about their biodiversity targets and present results not only based on the number of species, but also on their composition, which is significantly different from that of the primary forest baseline. While secondary forests are key to combating both the climate and the biodiversity crisis, their altered composition should not be overlooked.

In terms of primary forests, our results show that even disturbances that seemed to have minimal impact on diversity, such as selective logging, still led to significant shifts in community composition. In the Brazilian Amazon, selective logging is often promoted as a conservation tool and is allowed in certain protected areas and public forests. Our results show that this is not an innocuous activity and highlight the need for policies to be clear about the biodiversity impacts that selective logging can have on biodiversity. Fire and logging together have an even greater influence—logged‐and‐burned forests were as dissimilar to undisturbed forests as to secondary forests for all facets of diversity. Given that these forests present a lower alpha‐diversity and a significantly different tree community composition from undisturbed forests, it is expected that large areas of the Amazon may already be severely impoverished, and unable to provide the full range of ecosystem services found in undisturbed primary forests (Nunes et al. 2022). Our results reinforce the need to prevent wildfires across Amazonia as a way of slowing the biodiversity crisis.

Both large and small trees were affected by human disturbance, although effect sizes were greater in large trees for most alpha‐diversity comparisons. This is likely a consequence of recruitment time into each size cohort—trees will recruit faster to the smaller cohort (i.e., ≥ 2 cm DBH < 10 cm DBH) than in the larger one (i.e., ≥ 10 cm DBH). In human‐modified forests, decreases in alpha‐diversity can be partially offset by the recruitment of fast‐growing pioneer species (Cochrane and Schulze 1999; Laurance et al. 2011; Schwartz et al. 2014), which can reach larger sizes in ≤ 6 years (Mesquita et al. 2001). However, pioneers represent a smaller pool of species than that of old‐growth trees (Nascimento et al. 2005), thus not being able to completely compensate for the total number of species lost following forest modification. Furthermore, old‐growth tree species can take from decades to centuries to recruit to the larger cohort (i.e., ≥ 10 cm DB) (Ferreira et al. 2020; Vieira et al. 2005), maintaining lower levels of alpha‐diversity for a prolonged period of time. In terms of community composition, for all facets of diversity, large and small trees had similar sensitivity. Overall, our results indicate that human‐modified forests could remain significantly different from an undisturbed baseline for decades to come, especially given that the current smaller cohort holds a different set of species, suggesting that trees recruiting into the larger size will continue to be distinct from those found in undisturbed forests. Finally, another factor that could potentially contribute to human‐modified Amazonian forests holding altered tree communities for a prolonged period is the long lifespan of pioneer species, which live, on average, for 104 years (Laurance et al. 2004). The combination of very slow growth rates of old‐growth species and long lifespans of pioneers indicates that tree communities will remain altered for over a century, even if no new disturbances occur.

### 4.2 Which Form of Diversity Is the Most Sensitive to Human Modification?

Taxonomic diversity has been the basis of many questions in Ecology for almost a century (e.g., Clements 1936; Gleason 1926; Lindeman 1942). More recently, it was questioned whether functional and phylogenetic diversities would respond to stressors in the same way as taxonomic diversity (Faith 1992; Laureto et al. 2015). Here, when examining the impacts of human modification on alpha‐diversity, we found that the taxonomic and functional facets of diversity had similar sensitivity, while phylogenetic diversity was the least sensitive. It is likely that most lineages were negatively affected but still maintained representative species, explaining the observed patterns of phylogenetic alpha‐diversity and corroborating the idea of phylogenetic redundancy. Finally, all three diversity facets showed similar responses to human modification in terms of community composition. As such, when examining the impacts of human modification in Amazonian tree communities, the examination of taxonomic diversity seems sufficient to capture changes in both functional and phylogenetic diversities.

### 4.3 The Importance of Dominance Weighting for Diversity Assessment

Amazonian lowland forests are the most diverse on Earth, housing c. 16,000 tree species, or 22% of the world's total (Gatti et al. 2022; ter Steege et al. 2013). However, this hyperdiversity is not equally distributed among different taxa, with only 227 species accounting for almost half of all trees in the region (ter Steege et al. 2013). This pattern of hyperdominance could potentially be affected by human‐driven modification – something yet to be tested. Furthermore, dominance varies across forest strata, with different species dominating the canopy, the midstorey and the understorey (Draper et al. 2021). It remains unclear if human modification will affect dominance patterns differently in each stratum. Our results for alpha‐diversity show that the effects of human modification on rare, common and dominant species (i.e., *q*0, *q*1 and *q*2) are of similar magnitude, demonstrating that tree communities in human‐modified forests experience changes in evenness. For species composition, both common and dominant species (i.e., *q*1 and *q*2) were the most sensitive, indicating that the changes in the identity of these species are higher than for rare and less abundant ones in human‐modified forests. These alterations in community evenness and composition raise questions about the functioning of human‐modified forests—across undisturbed parts of the Amazon, hyperdominant species tend to have a disproportionately large contribution towards carbon storage and productivity (Fauset et al. 2015); thus changes in both the identity and the abundance of tree species could impact the carbon cycle.

## 5 Conclusion

Despite disturbed primary forests (i.e., logged, and logged‐and‐burned) having significantly lower levels of taxonomic, functional and phylogenetic diversity than undisturbed forests, they are still significantly more diverse than secondary forests. These results highlight two important and policy‐relevant points: (1) It is crucial to effectively protect undisturbed primary forests, avoiding human‐driven disturbance; and (2) It is essential to also protect disturbed primary forests from further human‐driven disturbances as they still hold high levels of diversity, with protection even more needed in places where there are few or no remnants of undisturbed forests. While recently there has been great investment and political interest in the restoration of Amazonian forests, our results clearly demonstrate that, for biodiversity conservation, we should prioritize the protection of undisturbed primary forests and avoid further disturbances in those already human‐modified.

## Author Contributions

**Erika Berenguer:** conceptualization, data curation, formal analysis, investigation, methodology, project administration, writing – original draft, writing – review and editing. **Cássio Alencar Nunes:** conceptualization, formal analysis, visualization, writing – original draft, writing – review and editing. **Luiz E. O. C. Aragão:** methodology, writing – review and editing. **Jesús Aguirre‐Gutiérrez:** formal analysis, writing – review and editing. **Joice Ferreira:** funding acquisition, methodology, project administration, supervision, writing – review and editing. **Yadvinder Malhi:** funding acquisition, methodology, writing – review and editing. **Adriane Esquivel‐Muelbert:** formal analysis, methodology, writing – review and editing. **Axa E. S. Figueiredo:** methodology, writing – review and editing. **Joseph E. Hawes:** formal analysis, methodology, writing – review and editing. **Carlos A. Joly:** funding acquisition, methodology, writing – review and editing. **Carlos A. Quesada:** methodology, writing – review and editing. **Marina M. M. de Seixas:** data curation, investigation, methodology, project administration. **Ima Vieira:** funding acquisition, methodology, project administration, writing – review and editing. **Jos Barlow:** conceptualization, funding acquisition, methodology, project administration, supervision, writing – original draft, writing – review and editing.

## Conflicts of Interest

The authors declare no conflicts of interest.

## Supporting information


**Data S1:** gcb70595‐sup‐0001‐Supinfo.pdf.

## Data Availability

The data and code that support the findings of this study are openly available in Zenodo at https://doi.org/10.5281/zenodo.17407989.
