# Supplementary material for: Multifaceted Assessment of Amazonian Tree Diversity Reveals Pervasive Impacts of Human Modification
Source: Glob Chang Biol. 2025 Nov 10;31(11):e70595. doi: 10.1111/gcb.70595 (PMC12598522; doi:10.1111/gcb.70595)
Supplement: Supplementary file 1 — Data S1: gcb70595‐sup‐0001‐Supinfo.pdf. [file GCB-31-e70595-s001.pdf]

SUPPLEMENTARY MATERIAL

Multi-faceted assessment of Amazonian tree diversity reveals pervasive impacts of human modification

Erika Berenguer* & Cássio A. Nunes*, Jesus Aguirre-Gutiérrez, Joice Ferreira, Yadvinder Malhi, Luiz E. O. C. Aragão, Adriane Esquivel-Muelbert, Axa E. S. Figueiredo, Carlos A. Joly, Carlos A. Quesada, Marina M. M. de Seixas, Ima Vieira, Jos Barlow

***** Shared first authorship

Contents

1 – Tables S1-S11

2 – Figures S1-S12

3 - References

Table S1 - The number of plots (250 x 10 m) sampled in each forest class in Paragominas and Santarém.

**Forest class**	**Paragominas**	**Santarém**
Undisturbed	13	17
Logged	44	26
Logged-and-burned	44	23
Secondary	16	32
**Total**	117	98

Table S2. List of all the 20 traits assessed, including their type (i.e. morphological or physiological) and importance.

Trait	Type	Importance	References
Bark thickness	Morphological	Thermal insulation, herbivore and pathogen protection	(Boland & Woodward, 2021; Brando et al., 2012; Franceschi et al., 2005)
Dispersal mode	Morphological	Dispersal, seedling survival	(Chen et al., 2023; Janzen, 1970)
Fruit type	Morphological	Dispersal, seed protection	(Herrera, 1995; Howe & Smallwood, 1982)
Leaf area	Morphological	Light capture	(Poorter et al., 2009; Rozendaal et al., 2006)
Leaf C	Physiological	Herbivory protection	(DeAngelis et al., 2012; Numata et al., 2003)
Leaf Ca	Physiological	Herbivory protection	(Franceschi & Nakata, 2005; Korth et al., 2006)
Leaf dry matter content	Morphological	Herbivory protection	(Bröcher et al., 2023)
Leaf Fe	Physiological	Photosynthetic capacity	(Spiller & Terry, 1980; Terry & Abadía, 1986)
Leaf K	Physiological	Stomatal control	(Santiago & Wright, 2007)
Leaf Mg	Physiological	Photosynthetic capacity	(Hermans et al., 2004; Shaul, 2002)
Leaf Mn	Physiological	Photosynthetic capacity	(Ivanov et al., 2022; Schmidt et al., 2016)
Leaf N	Physiological	Photosynthetic capacity	(Reich et al., 1994, 1997)
Leaf P	Physiological	Photosynthetic capacity	(Reich et al., 2009; Walker et al., 2014)
Leaf thickness	Morphological	Photosynthetic capacity, thermal insulation	(Niinemets, 1999; Vogel, 2009)
Leaf Zn	Physiological	Photosynthetic capacity	(Hänsch & Mendel, 2009; Mattiello et al., 2015)
Potential tree size	Morphological	Light access	(Falster & Westoby, 2003; Jackson et al., 2021)

Seed width	Morphological	Dispersal, germination success	(Jones et al., 2023; Levey, 1987)
Specific leaf area	Morphological	Growth rates, light capture, photosynthetic capacity	(Poorter et al., 2009; Reich et al., 1997)
Water deficit affiliation	Physiological	Drought tolerance	(Esquivel-Muelbert et al., 2017, 2019)
Wood density	Morphological	Structural support, water transport	(Chave et al., 2009; Hacke et al., 2001)

Table S3. Number and percentage of individuals in Paragominas with trait data assigned at the species, genus, or family level, as well as those without any data. For dispersal mode, fruit type, and seed width, trait values were taken from a source that attributed values from higher taxonomic levels to species without available data (Hawes et al., 2020), preventing disaggregation by taxonomic level.

Trait	Species-level data	Genus-level data	Family-level data	No data
Bark thickness	24123 (79%)	3749 (12%)	1282 (4%)	1500 (5%)
Dispersal mode	NA	NA	NA	977 (3%)
Fruit type	NA	NA	NA	638 (2%)
Leaf area	19771 (65%)	5319 (17%)	4161 (14%)	1401 (5%)
Leaf C	19559 (64%)	5423 (18%)	4271 (14%)	1401 (5%)
Leaf Ca	19466 (64%)	5516 (18%)	4271 (14%)	1401 (5%)
Leaf dry matter content	19773 (65%)	5319 (17%)	4161 (14%)	1401 (5%)
Leaf Fe	19625 (64%)	5441 (18%)	4187 (14%)	1401 (5%)
Leaf K	19756 (65%)	5311 (17%)	4186 (14%)	1401 (5%)
Leaf Mg	19672 (64%)	5310 (17%)	4271 (14%)	1401 (5%)
Leaf Mn	19613 (64%)	5369 (18%)	4271 (14%)	1401 (5%)
Leaf N	19540 (64%)	5420 (18%)	4289 (14%)	1405 (5%)
Leaf P	19756 (65%)	5311 (17%)	4186 (14%)	1401 (5%)
Leaf thickness	19773 (65%)	5319 (17%)	4161 (14%)	1401 (5%)
Leaf Zn	19712 (64%)	5349 (18%)	4192 (14%)	1401 (5%)
Potential tree size	24505 (80%)	4855 (16%)	1263 (4%)	31 (0.1%)
Seed width	NA	NA	NA	155 (0.5%)
Specific leaf area	19773 (65%)	5319 (17%)	4161 (14%)	1401 (5%)
Water deficit affiliation	18701 (61%)	11245 (37%)	688 (1%)	20 (<0.1%)
Wood density	20620 (67%)	9292 (30%)	740 (2%)	2 (<0.01%)

TableS4. Number and percentage of individuals in Santarém with trait data assigned at the species, genus, or family level, as well as those without any data. For dispersal mode, fruit type, and seed width, trait values were taken from a source that attributed values from higher taxonomic levels to species without available data (Hawes et al., 2020), preventing disaggregation by taxonomic level.

Trait	Species-level data	Genus-level data	Family-level data	No data
Bark thickness	19507 (79%)	2886 (12%)	1100 (5%)	1236 (5%)
Dispersal mode	NA	NA	NA	1030 (4%)
Fruit type	NA	NA	NA	214 (0.9%)
Leaf area	16184 (65%)	4179 (17%)	3624 (15%)	743 (3%)
Leaf C	16025 (65%)	4224 (17%)	3737 (15%)	743 (3%)
Leaf Ca	16085 (65%)	4216 (17%)	3685 (15%)	743 (3%)
Leaf dry matter content	16184 (66%)	4193 (17%)	3609 (15%)	743 (3%)
Leaf Fe	16025 (65%)	4309 (17%)	3652 (15%)	743 (3%)
Leaf K	16151 (65%)	4191 (17%)	3644 (15%)	743 (3%)
Leaf Mg	16110 (65%)	4191 (17%)	3685 (15%)	743 (3%)
Leaf Mn	16055 (65%)	4231 (17%)	3700 (15%)	743 (3%)
Leaf N	15991 (65%)	4213 (17%)	3777 (15%)	748 (3%)
Leaf P	16151 (65%)	4191 (17%)	3644 (15%)	743 (3%)
Leaf thickness	16184 (66%)	4193 (17%)	3609 (15%)	743 (3%)
Leaf Zn	16113 (65%)	4213 (17%)	3660 (15%)	743 (3%)
Potential tree size	18077 (73%)	5811 (24%)	793 (3%)	48 (<0.5%)
Seed width	NA	NA	NA	236 (1%)
Specific leaf area	16184 (66%)	4178 (17%)	3624 (15%)	743 (3%)
Water deficit affiliation	15591 (63%)	8452 (34%)	658 (3%)	28 (<0.5%)
Wood density	16640 (67%)	7706 (31%)	378 (2%)	5 (<0.05%)

Table S5. Estimated Marginal Means (EMM) of the effect sizes (from LMMs) of differences in alpha-diversity between forest class comparisons for taxonomic, functional and phylogenetic diversity. The EMMs are averaged over different forest class comparisons and over large and small trees. The significance column displays different letters for the diversity facets with significant statistical differences ($p < 0.05$).

Diversity facet	EMM	Significance
Taxonomic	0.880	a
Functional	0.818	a
Phylogenetic	0.615	b

Table S6. Estimated Marginal Means (EMM) of the effect sizes (from LMMs) of differences in alpha-diversity between forest class comparisons for large and small trees. The EMMs are averaged over taxonomic, functional and phylogenetic diversity. The significance column displays different letters for comparisons between size classes with significant statistical differences ($p < 0.05$), separated by each forest class comparison.

Forest class comparison	Tree size	EMM	Significance
Logged vs Secondary	Large	1.522	a
	Small	1.160	b
Undisturbed vs Secondary	Large	1.443	a
	Small	0.818	b
Logged-and-burned vs Secondary	Large	0.889	a
	Small	0.888	a
Logged vs Logged-and-burned	Large	0.665	a
	Small	0.354	b
Undisturbed vs Logged-and-burned	Large	0.561	a
	Small	0.328	b
Undisturbed vs Logged	Large	0.213	a
	Small	0.411	b

Table S7. Estimated Marginal Means (EMM) of the effect sizes (from LMMs) of differences in alpha-diversity between forest class comparisons for large and small trees. The EMMs are averaged over taxonomic, functional and phylogenetic diversity. The significance column displays different letters for forest class comparisons with significant statistical differences ($p < 0.05$).

Tree size	Forest class comparison	EMM	Significance
Large	Logged vs Secondary	1.522	a
	Undisturbed vs Secondary	1.443	a
	Logged-and-burned vs Secondary	0.889	b
	Logged vs Logged-and-burned	0.665	bc
	Undisturbed vs Logged-and-burned	0.561	c
	Undisturbed vs Logged	0.213	d
Small	Undisturbed vs Secondary	1.160	a
	Logged-and-burned vs Secondary	0.888	b
	Logged vs Secondary	0.818	b
	Undisturbed vs Logged	0.411	c
	Logged vs Logged-and-burned	0.354	c
	Undisturbed vs Logged-and-burned	0.328	c

Table S8. Estimated Marginal Means (EMM) of the effect sizes (from LMMs) of differences in alpha-diversity between forest classes comparisons for large and small trees and for taxonomic, functional, and phylogenetic diversity. The table is ordered from highest to lowest EMM values.

**Forest class comparison**	**Tree size**	**Diversity facet**	**EMM**
Logged vs Secondary	Large	Taxonomic	1.632
Logged vs Secondary	Large	Functional	1.570
Undisturbed vs Secondary	Large	Taxonomic	1.553
Undisturbed vs Secondary	Large	Functional	1.491
Logged vs Secondary	Large	Phylogenetic	1.367
Undisturbed vs Secondary	Large	Phylogenetic	1.288
Logged vs Secondary	Small	Taxonomic	1.270
Logged vs Secondary	Small	Functional	1.208
Logged vs Secondary	Small	Phylogenetic	1.005
Logged-and-burned vs Secondary	Large	Taxonomic	0.999
Logged-and-burned vs Secondary	Small	Taxonomic	0.998
Logged-and-burned vs Secondary	Large	Functional	0.937
Logged-and-burned vs Secondary	Small	Functional	0.935
Undisturbed vs Secondary	Small	Taxonomic	0.928
Undisturbed vs Secondary	Small	Functional	0.865
Logged vs Logged-and-burned	Large	Taxonomic	0.774
Logged-and-burned vs Secondary	Large	Phylogenetic	0.734
Logged-and-burned vs Secondary	Small	Phylogenetic	0.732
Logged vs Logged-and-burned	Large	Functional	0.712
Undisturbed vs Logged-and-burned	Large	Taxonomic	0.671
Undisturbed vs Secondary	Small	Phylogenetic	0.662
Undisturbed vs Logged-and-burned	Large	Functional	0.609
Undisturbed vs Logged	Small	Taxonomic	0.521
Logged vs Logged-and-burned	Large	Phylogenetic	0.509
Logged vs Logged-and-burned	Small	Taxonomic	0.463
Undisturbed vs Logged	Small	Functional	0.459
Undisturbed vs Logged-and-burned	Small	Taxonomic	0.437
Undisturbed vs Logged-and-burned	Large	Phylogenetic	0.406
Logged vs Logged-and-burned	Small	Functional	0.401
Undisturbed vs Logged-and-burned	Small	Functional	0.375
Undisturbed vs Logged	Large	Taxonomic	0.322
Undisturbed vs Logged	Large	Functional	0.260
Undisturbed vs Logged	Small	Phylogenetic	0.256
Logged vs Logged-and-burned	Small	Phylogenetic	0.198
Undisturbed vs Logged-and-burned	Small	Phylogenetic	0.172

Undisturbed vs Logged	Large	Phylogenetic	0.057

Table S9. Estimated Marginal Means (EMM) of the effect sizes (Omega² from PERMANOVAs) of differences in community composition from comparisons between forest classes using different dominance weightings (i.e., q0, q1, and q2). The EMMs are averaged over different forest class comparisons and over large and small trees. The significance column displays different letters for the dominance weightings with statistical differences ($p < 0.05$).

**Dominance weighting**	**EMM**	**Significance**
q0	0.111	a
q1	0.165	b
q2	0.156	b

Table S10. Estimated Marginal Means (EMM) of the effect sizes (Omega² from PERMANOVAs) of differences in community composition from comparisons between forest classes. The EMMs are averaged over large and small trees and over different dominance weightings (i.e., q0, q1, and q2). The significance column displays different letters for forest class comparisons with significant statistical differences ($p < 0.05$).

**Forest class comparison**	**EMM**	**Significance**
Undisturbed vs Secondary	0.287	a
Logged vs Secondary	0.213	b
Undisturbed vs Logged-and-burned	0.108	c
Logged-and-burned vs Secondary	0.101	cd
Undisturbed vs Logged	0.094	cd
Logged vs Logged-and-burned	0.062	d

Table S11. Estimated Marginal Means (EMM) of the effect sizes (Omega² from PERMANOVAs) of differences in community composition between forest class comparisons for large and small trees using different dominance weightings (i.e., q0, q1, and q2). The table is ordered from highest to lowest EMM values.

Forest class comparison	Dominance weighting	EMM
Undisturbed vs Secondary	q1	0.308
Undisturbed vs Secondary	q2	0.298
Undisturbed vs Secondary	q0	0.254
Logged vs Secondary	q1	0.234
Logged vs Secondary	q2	0.224
Logged vs Secondary	q0	0.180
Undisturbed vs Logged-and-burned	q1	0.129
Logged-and-burned vs Secondary	q1	0.122
Undisturbed vs Logged-and-burned	q2	0.119
Undisturbed vs Logged	q1	0.115
Logged-and-burned vs Secondary	q2	0.112
Undisturbed vs Logged	q2	0.106
Logged vs Logged-and-burned	q1	0.083
Undisturbed vs Logged-and-burned	q0	0.075
Logged vs Logged-and-burned	q2	0.073
Logged-and-burned vs Secondary	q0	0.068
Undisturbed vs Logged	q0	0.062
Logged vs Logged-and-burned	q0	0.029

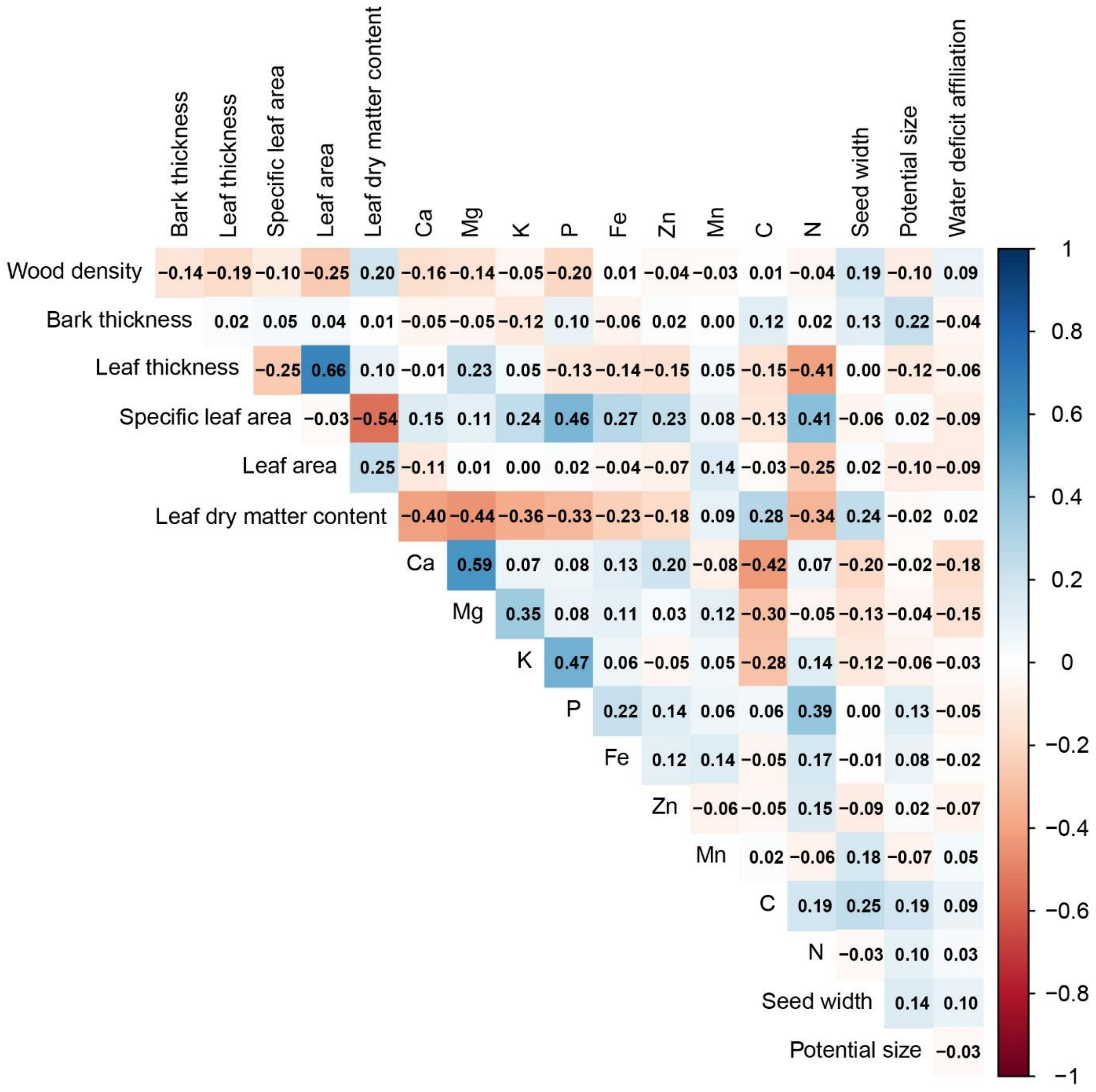

Figure S1. Correlation matrix of all continuous morphological and chemical functional traits of Amazonian trees (n=17 traits). Values represent the coefficient of correlation of Pearson (-1 to 1).

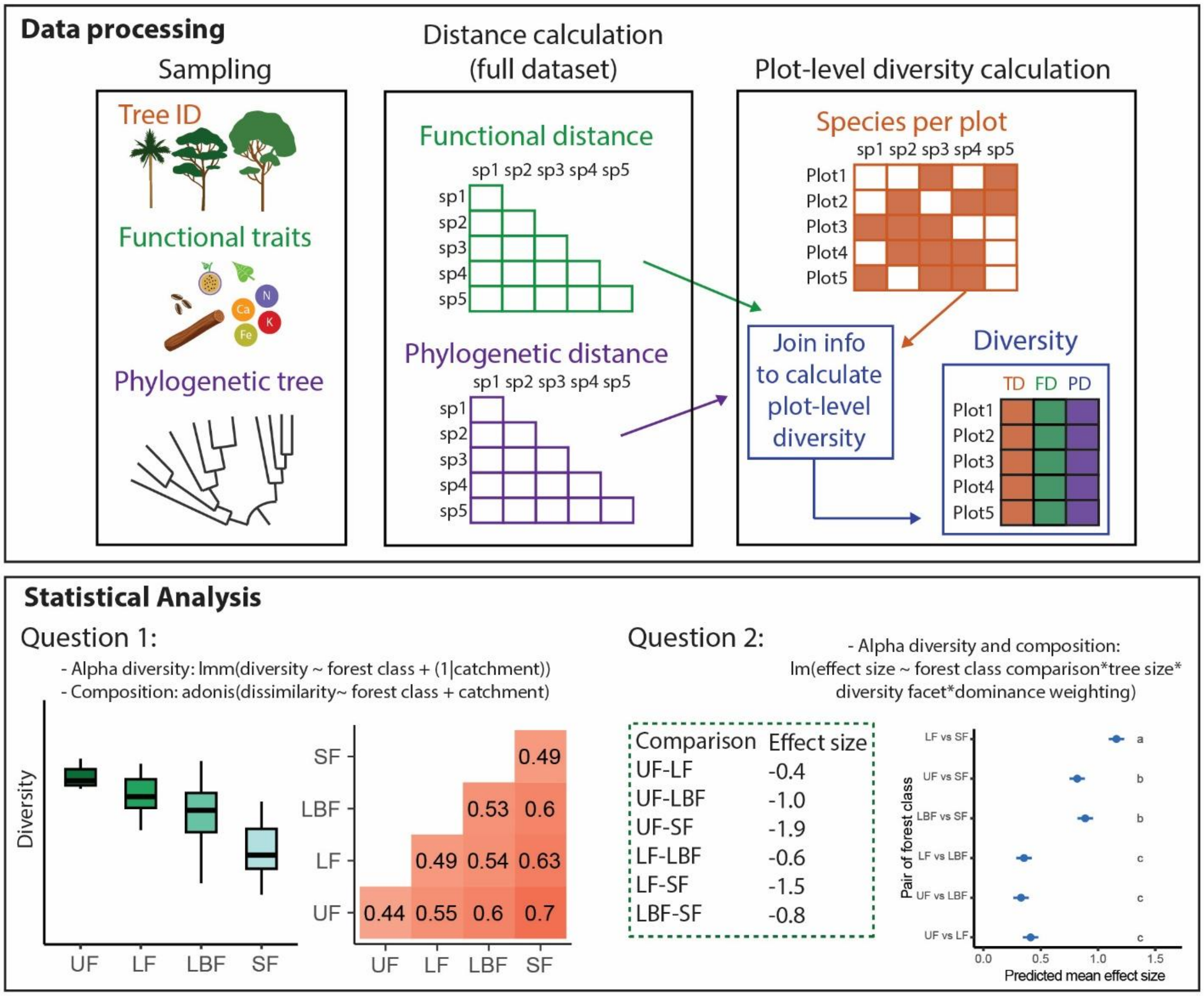

Figure S2. Analysis protocol. The top box of the figure shows the protocols for data processing before conducting the statistical analysis, which in turn are shown in the bottom box. The first step, following the field sampling, is to calculate functional and phylogenetic distances between all species in the full dataset. The third step is to join the information of which species is present in each plot with their functional and phylogenetic distance information to calculate plot-level Taxonomic, Functional and Phylogenetic diversities (TD, FD, PD). With plot-level TD, FD and PD, we can statistically test if diversity differs in different forest classes (Question 1). We can then estimate effect sizes for each forest class comparison and test which facet of diversity is more sensitive to human-induced disturbance (Question 2).

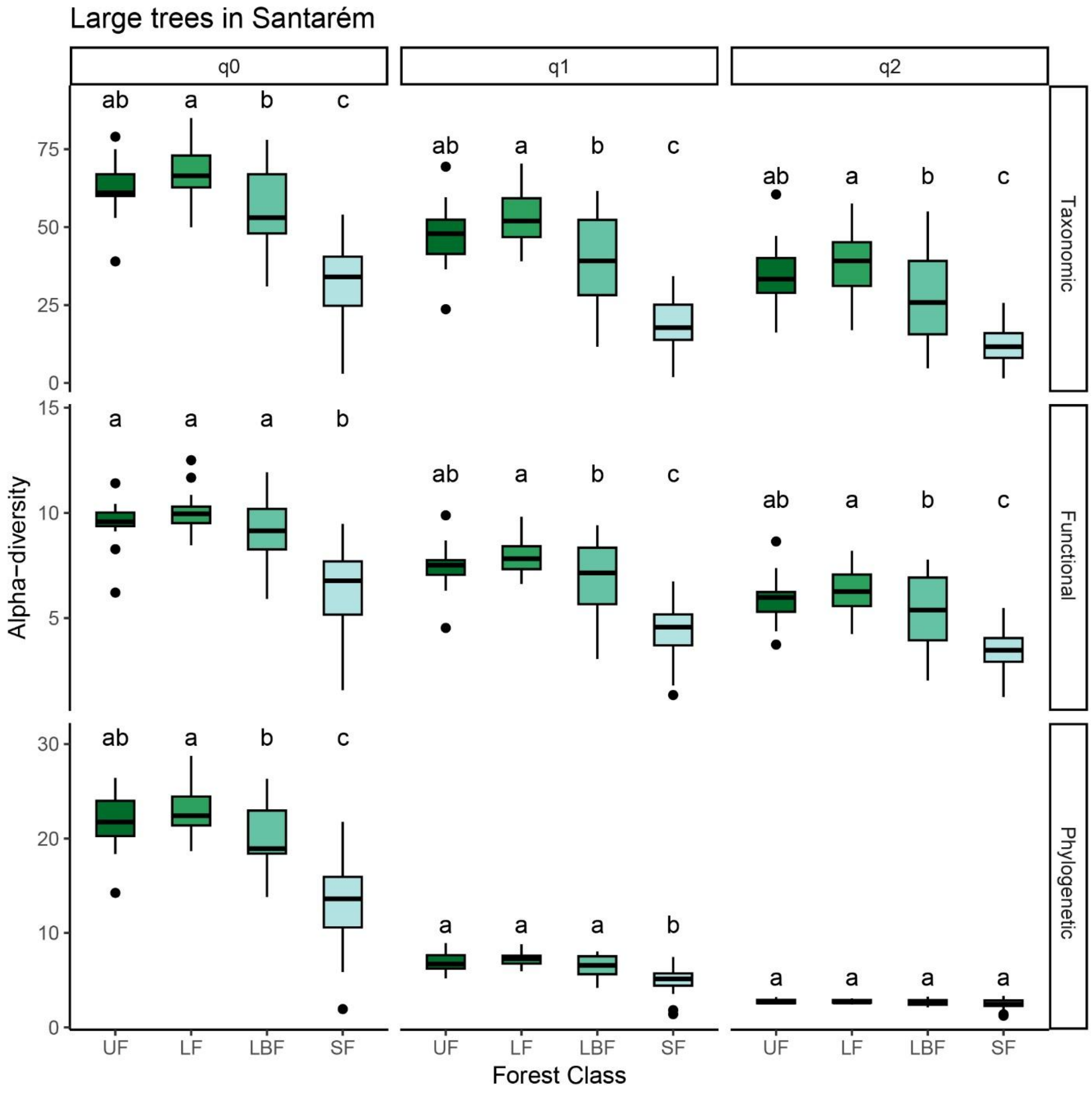

Figure S3. Alpha-diversity of large trees sampled in four forest classes in Santarém, in the Brazilian Amazon. Alpha-diversity was calculated for each dominance weighting (i.e., q0, q1, and q2) and diversity facet. The Y axis for taxonomic, functional and phylogenetic diversity have different scales. Different letters denote significant differences ($p < 0.05$) in mean values separately for each dominance weighting and diversity facet based on LMMs (see Methods). UF = undisturbed forests, LF = logged forests, LBF = logged-and-burned forests, SF = secondary forests.

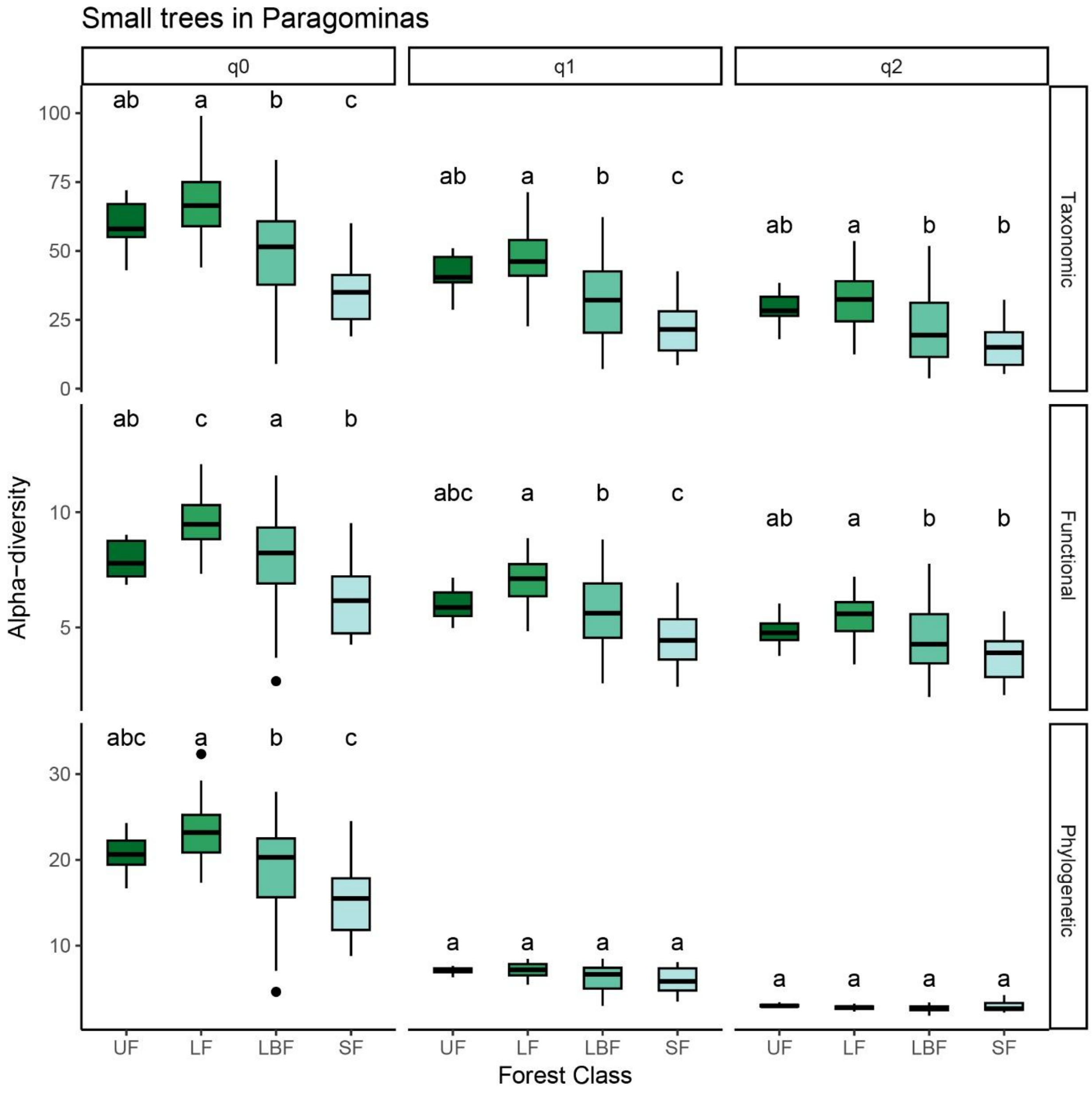

Figure S4. Alpha-diversity of small trees sampled in four forest classes in Paragominas, in the Brazilian Amazon. Alpha-diversity was calculated for each dominance weighting (i.e., q0, q1, and q2) and diversity facet. The Y axis for taxonomic, functional and phylogenetic diversity have different scales. Different letters denote significant differences ($p < 0.05$) in mean values separately for each dominance weighting and diversity facet based on LMMs (see Methods). UF = undisturbed forests, LF = logged forests, LBF = logged-and-burned forests, SF = secondary forests.

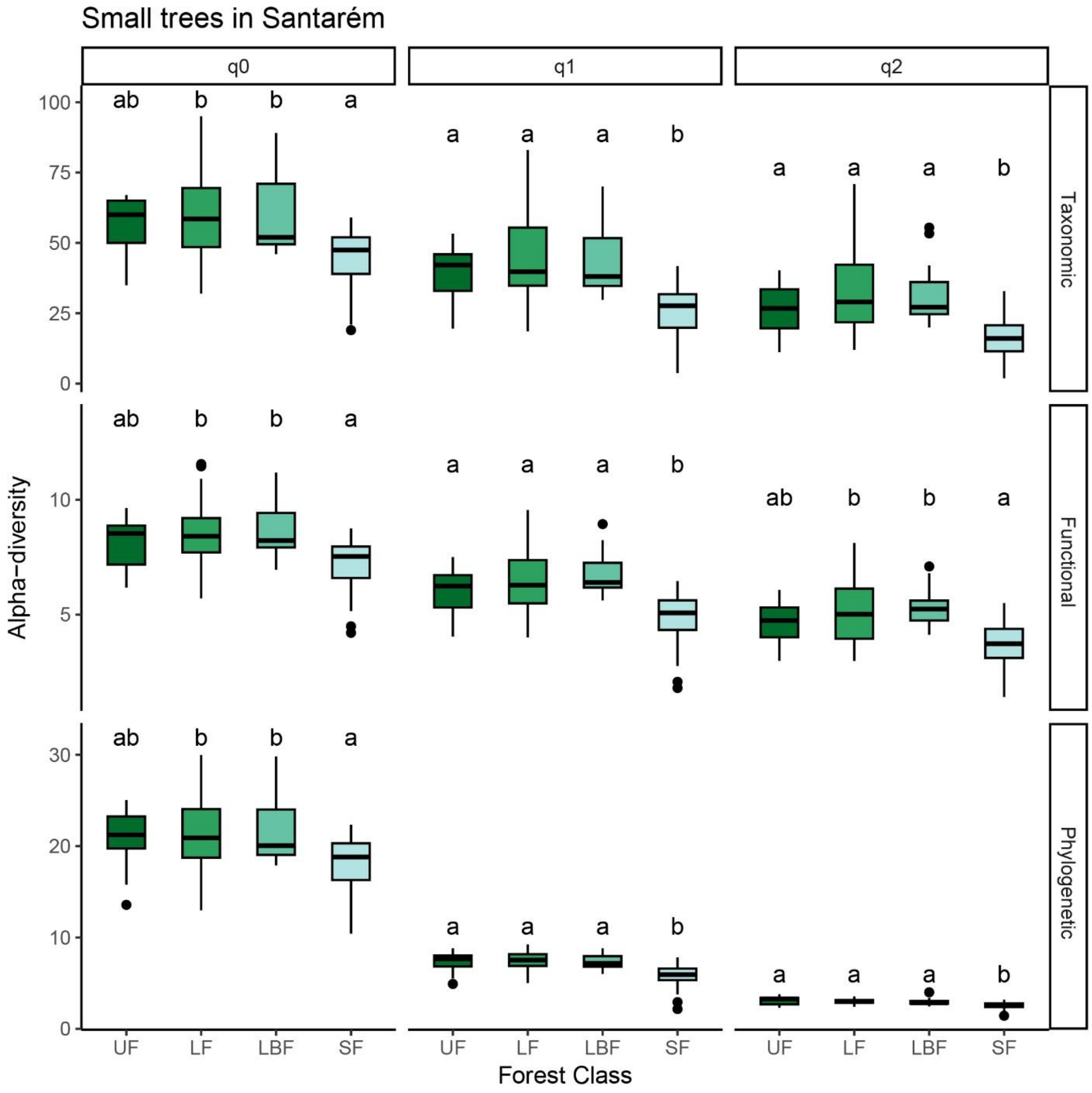

Figure S5. Alpha-diversity of small trees sampled in four forest classes in Santarém, in the Brazilian Amazon. Alpha-diversity was calculated for each dominance weighting (i.e., q0, q1, and q2) and diversity facet. The Y axis for taxonomic, functional and phylogenetic diversity have different scales. Different letters denote significant differences ($p < 0.05$) in mean values separately for each dominance weighting and diversity facet based on LMMs (see Methods). UF = undisturbed forests, LF = logged forests, LBF = logged-and-burned forests, SF = secondary forests.

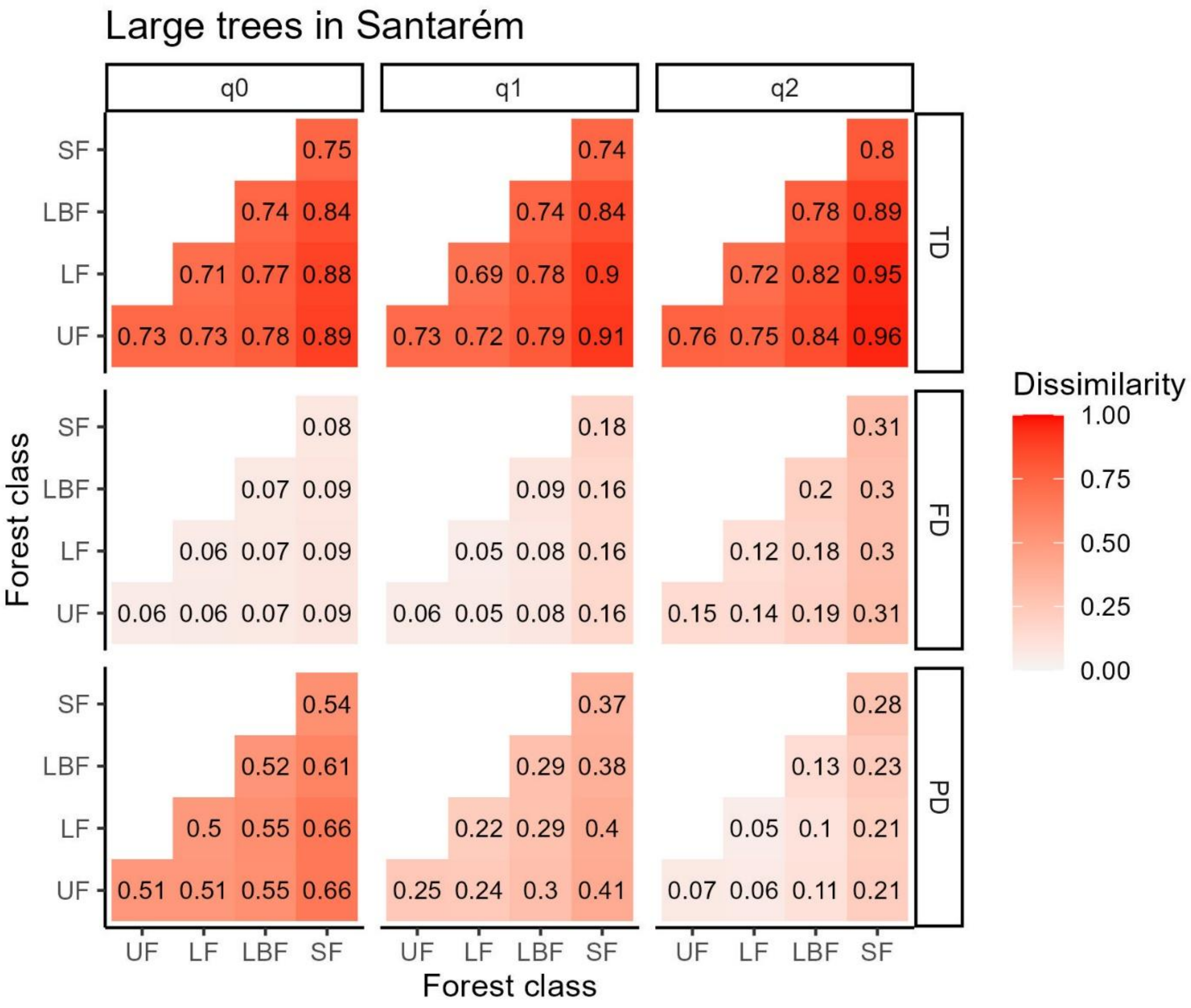

Figure S6. Mean pairwise dissimilarity in community composition of large trees sampled in four forest classes in Santarém, in the Brazilian Amazon. Dissimilarity was calculated using different dominance weightings (i.e., q0, q1 and q2) for taxonomic, functional and phylogenetic diversity. UF = undisturbed forests, LF = logged forests, LBF = logged-and-burned forests, SF = secondary forests.

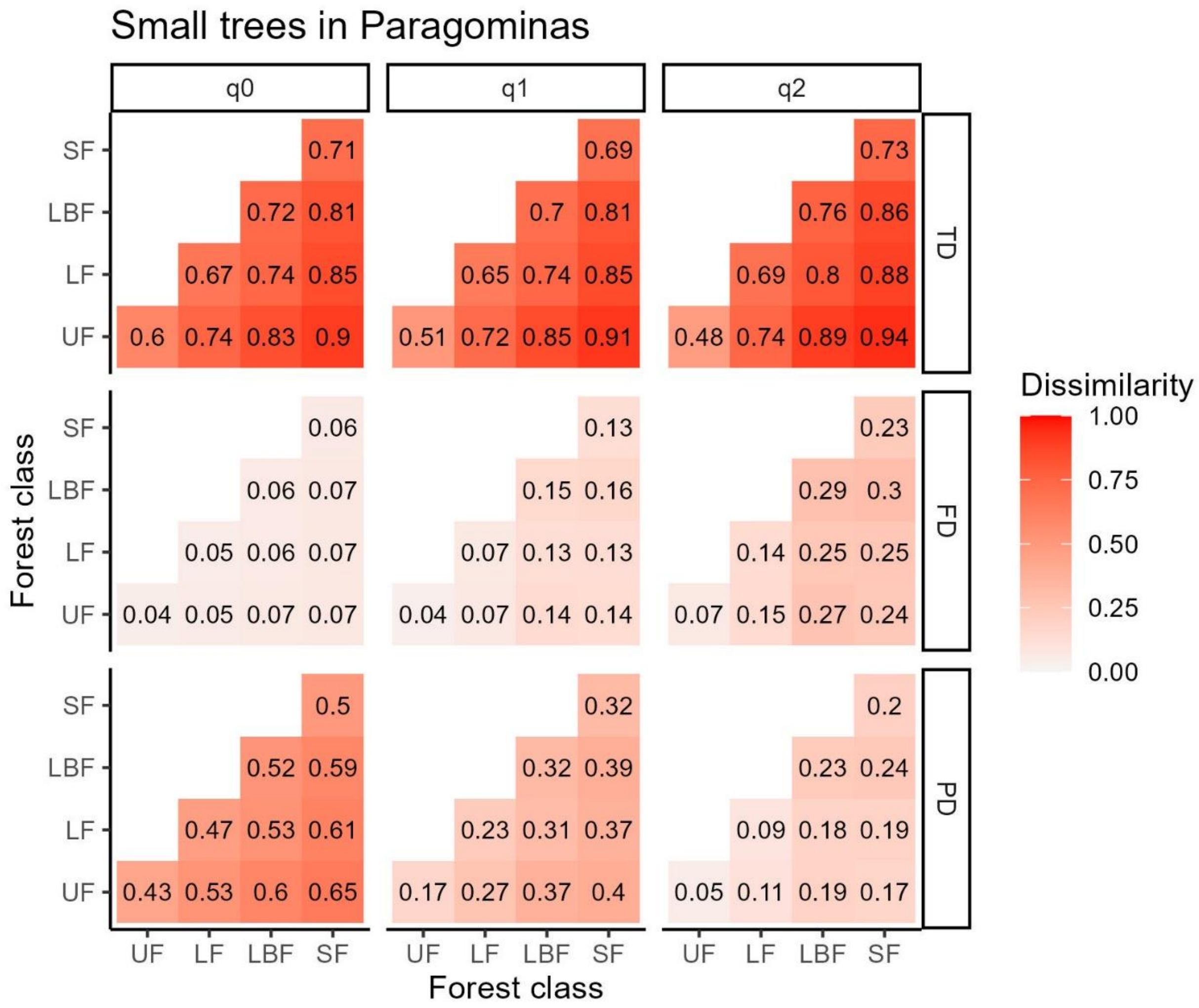

Figure S7. Mean pairwise dissimilarity in community composition of small trees sampled in four forest classes in Paragominas, in the Brazilian Amazon. Dissimilarity was calculated using different dominance weightings (i.e., q0, q1 and q2) for taxonomic, functional and phylogenetic diversity. UF = undisturbed forests, LF = logged forests, LBF = logged-and-burned forests, SF = secondary forests.

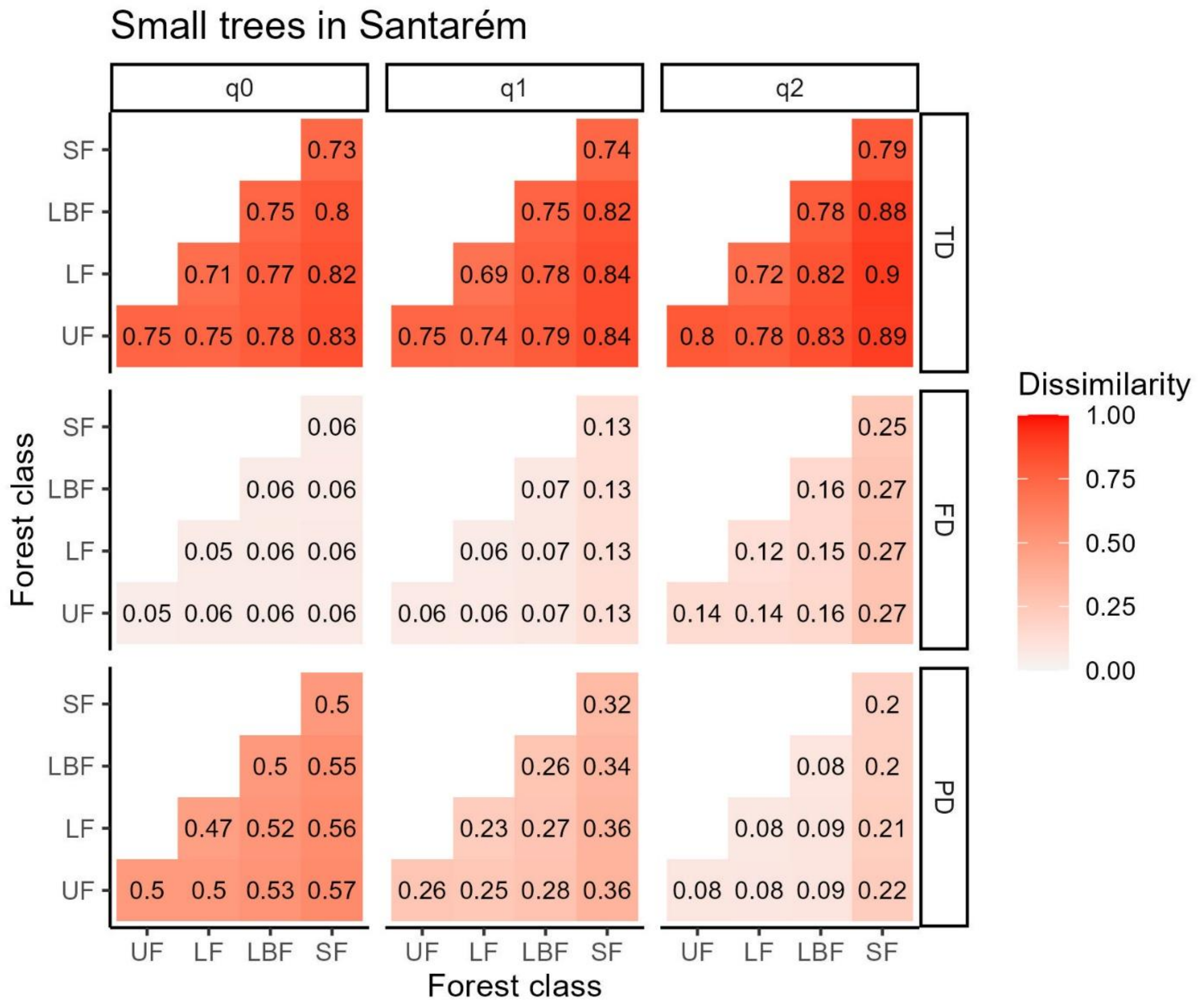

Figure S8. Mean pairwise dissimilarity in community composition of small trees sampled in four forest classes in Santarém, in the Brazilian Amazon. Dissimilarity was calculated using different dominance weightings (i.e., q0, q1 and q2) for taxonomic, functional, and phylogenetic diversity. UF = undisturbed forests, LF = logged forests, LBF = logged-and-burned forests, SF = secondary forests.

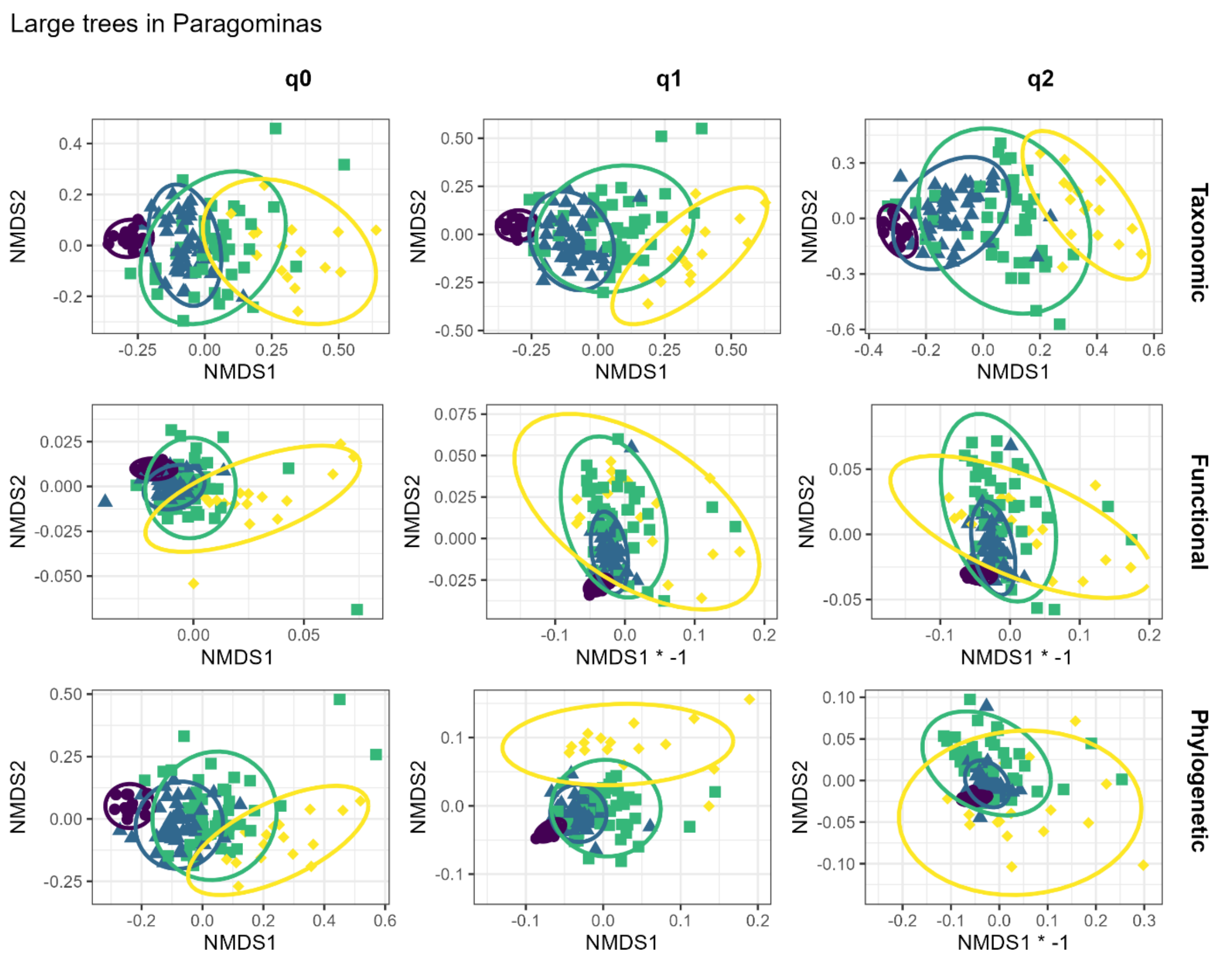

Figure S9. Graphical representation of community composition (NMDS) of large trees sampled in four forest classes in Paragominas, in the Brazilian Amazon. Dissimilarity was calculated using different dominance weightings (i.e., q0, q1 and q2) for taxonomic, functional, and phylogenetic diversity. Ellipses represent the 95% confidence interval of the centroid for each forest class. All data was used in the NMDS analysis and ellipses calculation, but we excluded outliers from the graphs to ease the visualization of patterns. Undisturbed forests = purple, Logged forests = blue, Logged-and-burned forests = green, Secondary forests = yellow.

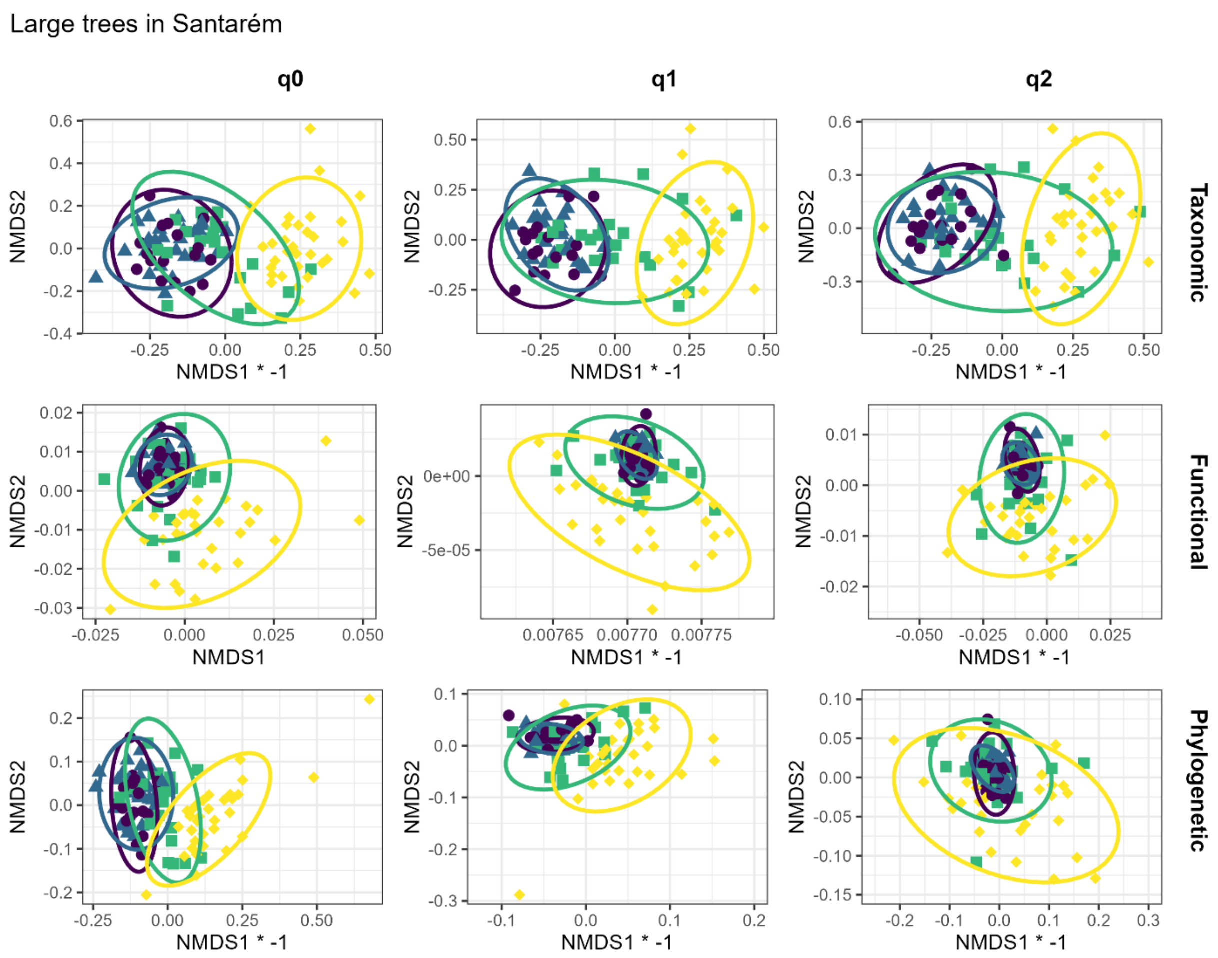

Figure S10. Graphical representation of community composition (NMDS) of large trees sampled in four forest classes in Santarém, in the Brazilian Amazon. Dissimilarity was calculated using different dominance weightings (i.e., q0, q1 and q2) for taxonomic, functional, and phylogenetic diversity. Ellipses represent the 95% confidence interval of the centroid for each forest class. All data was used in the NMDS analysis and ellipses calculation, but we excluded outliers from the graphs to ease the visualization of patterns. Undisturbed forests = purple, Logged forests = blue, Logged-and-burned forests = green, Secondary forests = yellow.

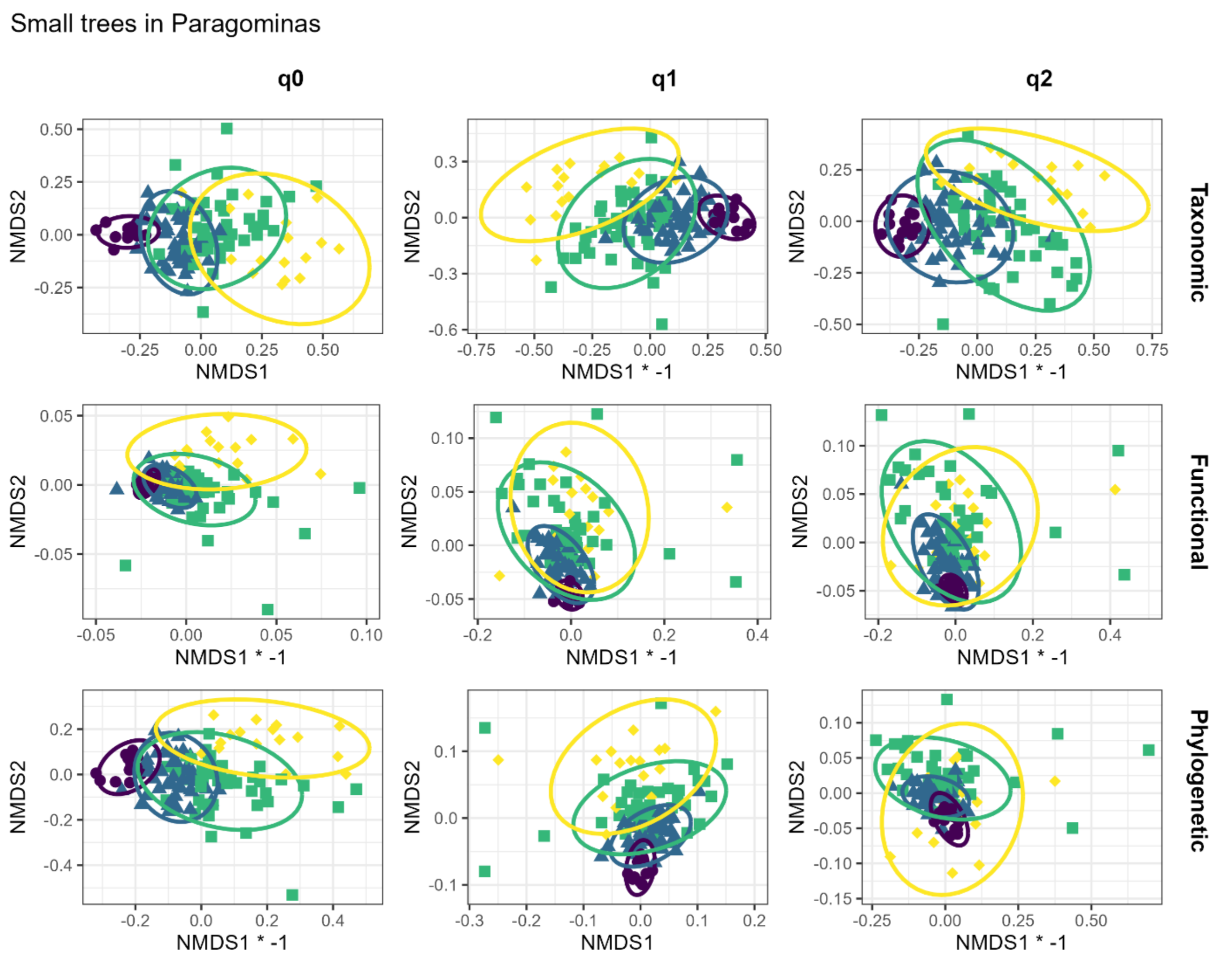

Figure S11. Graphical representation of community composition (NMDS) of small trees sampled in four forest classes in Paragominas, in the Brazilian Amazon. Dissimilarity was calculated using different dominance weightings (i.e., q0, q1 and q2) for taxonomic, functional, and phylogenetic diversity. Ellipses represent the 95% confidence interval of the centroid for each forest class. All data was used in the NMDS analysis and ellipses calculation, but we excluded outliers from the graphs to ease the visualization of patterns. Undisturbed forests = purple, Logged forests = blue, Logged-and-burned forests = green, Secondary forests = yellow.

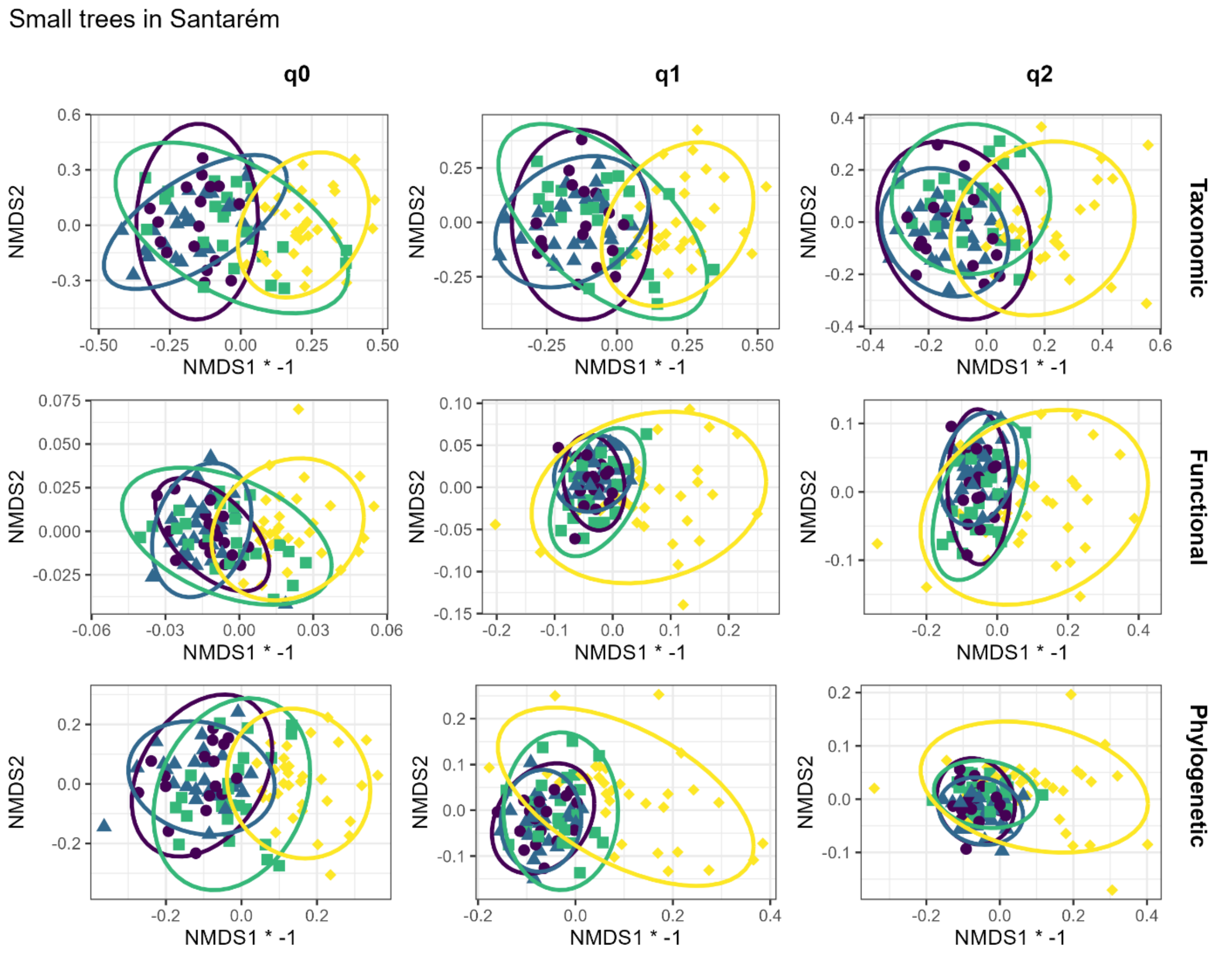

Figure S12. Graphical representation of community composition (NMDS) of small trees sampled in four forest classes in Santarém, in the Brazilian Amazon. Dissimilarity was calculated using different dominance weightings (i.e., q0, q1 and q2) for taxonomic, functional, and phylogenetic diversity. Ellipses represent the 95% confidence interval of the centroid for each forest class. All data was used in the NMDS analysis and ellipses calculation, but we excluded outliers from the graphs to ease the visualization of patterns. Undisturbed forests = purple, Logged forests = blue, Logged-and-burned forests = green, Secondary forests = yellow.

References

Boland, J. M., & Woodward, D. L. (2021). Thick bark can protect trees from a severe ambrosia beetle attack. *PeerJ*, *9*, e10755. https://doi.org/10.7717/peerj.10755

Brando, P. M., Nepstad, D. C., Balch, J. K., Bolker, B., Christman, M. C., Coe, M., & Putz, F. E. (2012). Fire-induced tree mortality in a neotropical forest: The roles of bark traits, tree size, wood density and fire behavior. *Global Change Biology*, *18*(2), 630–641. https://doi.org/10.1111/j.1365-2486.2011.02533.x

Bröcher, M., Ebeling, A., Hertzog, L., Roscher, C., Weisser, W., & Meyer, S. T. (2023). Effects of plant diversity on species-specific herbivory: Patterns and mechanisms. *Oecologia*, *201*(4), 1053–1066. https://doi.org/10.1007/s00442-023-05361-6

Chave, J., Coomes, D., Jansen, S., Lewis, S. L., Swenson, N. G., & Zanne, A. E. (2009). Towards a worldwide wood economics spectrum. *Ecology Letters*, *12*(4), 351–366. https://doi.org/10.1111/j.1461-0248.2009.01285.x

Chen, Y.-S., Muellner-Riehl, A. N., Yang, Y., Liu, J., Dimitrov, D., Luo, A., Luo, Y., Sun, H., & Wang, Z.-H. (2023). Dispersal modes affect Rhamnaceae diversification rates in a differentiated manner. *Proceedings of the Royal Society B: Biological Sciences*, *290*(2011), 20231926. https://doi.org/10.1098/rspb.2023.1926

DeAngelis, D. L., Ju, S., Liu, R., Bryant, J. P., & Gourley, S. A. (2012). Plant allocation of carbon to defense as a function of herbivory, light and nutrient availability. *Theoretical Ecology*, *5*(3), 445–456. https://doi.org/10.1007/s12080-011-0135-z

Esquivel-Muelbert, A., Baker, T. R., Dexter, K. G., Lewis, S. L., Brienen, R. J. W., Feldpausch, T. R., Lloyd, J., Monteagudo-Mendoza, A., Arroyo, L., Álvarez-Dávila, E., Higuchi, N., Marimon, B. S., Marimon-Junior, B. H., Silveira, M., Vilanova, E., Gloor, E., Malhi, Y.,

Chave, J., Barlow, J., … Phillips, O. L. (2019). Compositional response of Amazon forests to climate change. *Global Change Biology*, *25*(1), 39–56. https://doi.org/10.1111/gcb.14413

Esquivel-Muelbert, A., Galbraith, D., Dexter, K. G., Baker, T. R., Lewis, S. L., Meir, P., Rowland, L., Costa, A. C. L. da, Nepstad, D., & Phillips, O. L. (2017). Biogeographic distributions of neotropical trees reflect their directly measured drought tolerances. *Scientific Reports*, *7*(1), 8334. https://doi.org/10.1038/s41598-017-08105-8

Falster, D. S., & Westoby, M. (2003). Plant height and evolutionary games. *Trends in Ecology & Evolution*, *18*(7), 337–343. https://doi.org/10.1016/S0169-5347(03)00061-2

Franceschi, V. R., Krokene, P., Christiansen, E., & Krekling, T. (2005). Anatomical and chemical defenses of conifer bark against bark beetles and other pests. *New Phytologist*, *167*(2), 353–376. https://doi.org/10.1111/j.1469-8137.2005.01436.x

Franceschi, V. R., & Nakata, P. A. (2005). CALCIUM OXALATE IN PLANTS: Formation and Function. *Annual Review of Plant Biology*, *56*(Volume 56, 2005), 41–71. https://doi.org/10.1146/annurev.arplant.56.032604.144106

Hacke, U. G., Sperry, J. S., Pockman, W. T., Davis, S. D., & McCulloh, K. A. (2001). Trends in wood density and structure are linked to prevention of xylem implosion by negative pressure. *Oecologia*, *126*(4), 457–461. https://doi.org/10.1007/s004420100628

Hänsch, R., & Mendel, R. R. (2009). Physiological functions of mineral micronutrients (Cu, Zn, Mn, Fe, Ni, Mo, B, Cl). *Current Opinion in Plant Biology*, *12*(3), 259–266. https://doi.org/10.1016/j.pbi.2009.05.006

Hawes, J. E., Vieira, I. C. G., Magnago, L. F. S., Berenguer, E., Ferreira, J., Aragão, L. E. O. C., Cardoso, A., Lees, A. C., Lennox, G. D., Tobias, J. A., Waldron, A., & Barlow, J. (2020).

A large-scale assessment of plant dispersal mode and seed traits across human-modified Amazonian forests. *Journal of Ecology*, 1365-2745.13358. https://doi.org/10.1111/1365-2745.13358

Hermans, C., Johnson, G. N., Strasser, R. J., & Verbruggen, N. (2004). Physiological characterisation of magnesium deficiency in sugar beet: Acclimation to low magnesium differentially affects photosystems I and II. *Planta*, *220*(2), 344–355. https://doi.org/10.1007/s00425-004-1340-4

Herrera, C. M. (1995). Plant-Vertebrate Seed Dispersal Systems in the Mediterranean: Ecological, Evolutionary, and Historical Determinants. *Annual Review of Ecology and Systematics*, *26*, 705–727.

Howe, H. F., & Smallwood, J. (1982). Ecology of Seed Dispersal. *Annual Review of Ecology and Systematics*, *13*, 201–228.

Ivanov, Y. V., Pashkovskiy, P. P., Ivanova, A. I., Kartashov, A. V., & Kuznetsov, V. V. (2022). Manganese Deficiency Suppresses Growth and Photosynthetic Processes but Causes an Increase in the Expression of Photosynthetic Genes in Scots Pine Seedlings. *Cells*, *11*(23), 3814. https://doi.org/10.3390/cells11233814

Jackson, T. D., Shenkin, A. F., Majalap, N., Bin Jami, J., Bin Sailim, A., Reynolds, G., Coomes, D. A., Chandler, C. J., Boyd, D. S., Burt, A., Wilkes, P., Disney, M., & Malhi, Y. (2021). The mechanical stability of the world's tallest broadleaf trees. *Biotropica*, *53*(1), 110–120. https://doi.org/10.1111/btp.12850

Janzen, D. H. (1970). Herbivores and the Number of Tree Species in Tropical Forests. *The American Naturalist*, *104*(940), 501–528.

Jones, L. R., Hunts, C. A., Dolan, L. A., Murphy, N. K., Ripa, G. N., Schultz, E. A., Shastry, V. S., Sklarczyk, C. A., Thornton, B. S., & Boudreau, M. R. (2023). Effects of seed size and toucan regurgitation on the germination of the tropical tree Eugenia uniflora. *Journal of Tropical Ecology*, *39*, e5. https://doi.org/10.1017/S026646742200044X

Korth, K. L., Doege, S. J., Park, S.-H., Goggin, F. L., Wang, Q., Gomez, S. K., Liu, G., Jia, L., & Nakata, P. A. (2006). Medicago truncatula Mutants Demonstrate the Role of Plant Calcium Oxalate Crystals as an Effective Defense against Chewing Insects. *Plant Physiology*, *141*(1), 188–195. https://doi.org/10.1104/pp.106.076737

Levey, D. J. (1987). Seed Size and Fruit-Handling Techniques of Avian Frugivores. *The American Naturalist*, *129*(4), 471–485. https://doi.org/10.1086/284652

Mattiello, E. M., Ruiz, H. A., Neves, J. C. L., Ventrella, M. C., & Araújo, W. L. (2015). Zinc deficiency affects physiological and anatomical characteristics in maize leaves. *Journal of Plant Physiology*, *183*, 138–143. https://doi.org/10.1016/j.jplph.2015.05.014

Niinemets, Ü. (1999). Research review. Components of leaf dry mass per area – thickness and density – alter leaf photosynthetic capacity in reverse directions in woody plants. *New Phytologist*, *144*(1), 35–47. https://doi.org/10.1046/j.1469-8137.1999.00466.x

Numata, S., Kachi, N., Okuda, T., & Manokaran, N. (2003). Leaf Herbivory and Defenses of Dipterocarp Seedlings in the Pasoh Forest Reserve. In T. Okuda, N. Manokaran, Y. Matsumoto, K. Niiyama, S. C. Thomas, & P. S. Ashton (Eds.), *Pasoh: Ecology of a Lowland Rain Forest in Southeast Asia* (pp. 495–505). Springer Japan. https://doi.org/10.1007/978-4-431-67008-7_34

Poorter, H., Niinemets, Ü., Poorter, L., Wright, I. J., & Villar, R. (2009). Causes and consequences of variation in leaf mass per area (LMA): A meta-analysis. *New Phytologist*, *182*(3), 565–588. https://doi.org/10.1111/j.1469-8137.2009.02830.x

Reich, P. B., Oleksyn, J., & Wright, I. J. (2009). Leaf phosphorus influences the photosynthesis–nitrogen relation: A cross-biome analysis of 314 species. *Oecologia*, *160*(2), 207–212. https://doi.org/10.1007/s00442-009-1291-3

Reich, P. B., Walters, M. B., & Ellsworth, D. S. (1997). From tropics to tundra: Global convergence in plant functioning. *Proceedings of the National Academy of Sciences*, *94*(25), 13730–13734. https://doi.org/10.1073/pnas.94.25.13730

Reich, P. B., Walters, M. B., Ellsworth, D. S., & Uhl, C. (1994). Photosynthesis-nitrogen relations in Amazonian tree species. *Oecologia*, *97*(1), 62–72. https://doi.org/10.1007/BF00317909

Rozendaal, D. M. A., Hurtado, V. H., & Poorter, L. (2006). Plasticity in leaf traits of 38 tropical tree species in response to light; relationships with light demand and adult stature. *Functional Ecology*, *20*(2), 207–216. https://doi.org/10.1111/j.1365-2435.2006.01105.x

Santiago, L. S., & Wright, S. J. (2007). Leaf Functional Traits of Tropical Forest Plants in Telation to Growth Form. *Functional Ecology*, *21*(1), 19–27.

Schmidt, S. B., Jensen, P. E., & Husted, S. (2016). Manganese Deficiency in Plants: The Impact on Photosystem II. *Trends in Plant Science*, *21*(7), 622–632. https://doi.org/10.1016/j.tplants.2016.03.001

Shaul, O. (2002). Magnesium transport and function in plants: The tip of the iceberg. *Biometals*, *15*(3), 307–321. https://doi.org/10.1023/A:1016091118585

Spiller, S., & Terry, N. (1980). Limiting Factors in Photosynthesis: II. IRON STRESS DIMINISHES PHOTOCHEMICAL CAPACITY BY REDUCING THE NUMBER OF PHOTOSYNTHETIC UNITS 1 2. *Plant Physiology*, *65*(1), 121–125. https://doi.org/10.1104/pp.65.1.121

Terry, N., & Abadía, J. (1986). Function of iron in chloroplasts. *Journal of Plant Nutrition*, *9*(3–7), 609–646. https://doi.org/10.1080/01904168609363470

Vogel, S. (2009). Leaves in the lowest and highest winds: Temperature, force and shape. *New Phytologist*, *183*(1), 13–26. https://doi.org/10.1111/j.1469-8137.2009.02854.x

Walker, A. P., Beckerman, A. P., Gu, L., Kattge, J., Cernusak, L. A., Domingues, T. F., Scales, J. C., Wohlfahrt, G., Wullschleger, S. D., & Woodward, F. I. (2014). The relationship of leaf photosynthetic traits – Vcmax and Jmax – to leaf nitrogen, leaf phosphorus, and specific leaf area: A meta-analysis and modeling study. *Ecology and Evolution*, *4*(16), 3218–3235. https://doi.org/10.1002/ece3.1173
